# Transmembrane TNF drives osteoproliferative joint inflammation reminiscent of human spondyloarthritis

**DOI:** 10.1084/jem.20200288

**Published:** 2020-07-14

**Authors:** Merlijn H. Kaaij, Melissa N. van Tok, Iris C. Blijdorp, Carmen A. Ambarus, Michael Stock, Désiree Pots, Véronique L. Knaup, Marietta Armaka, Eleni Christodoulou-Vafeiadou, Tessa K. van Melsen, Huriatul Masdar, Harry J.P.P. Eskes, Nataliya G. Yeremenko, George Kollias, Georg Schett, Sander W. Tas, Leonie M. van Duivenvoorde, Dominique L.P. Baeten

**Affiliations:** 1 Amsterdam Rheumatology and Immunology Center, Department of Clinical Immunology and Rheumatology, Amsterdam University Medical Centers, University of Amsterdam, Amsterdam, Netherlands; 2 Department of Experimental Immunology, Amsterdam University Medical Centers, University of Amsterdam, Amsterdam, Netherlands; 3 Medizinische Klinik 3 - Rheumatologie und Immunologie, Universitätsklinikum Erlangen, Friedrich-Alexander-Universität Erlangen-Nürnberg, Erlangen, Germany; 4 Division of Immunology, Biomedical Sciences Research Center “Alexander Fleming,” Vari, Greece; 5 Department of Physiology, Medical School, National and Kapodistrian University of Athens, Athens, Greece; 6 Biomedcode Hellas Société Anonyme, Vari, Greece; 7 Department of Radiology, Amsterdam UMC, University of Amsterdam, Amsterdam, Netherlands

## Abstract

TNF plays a key role in immune-mediated inflammatory diseases including rheumatoid arthritis (RA) and spondyloarthritis (SpA). It remains incompletely understood how TNF can lead to different disease phenotypes such as destructive peripheral polysynovitis in RA versus axial and peripheral osteoproliferative inflammation in SpA. We observed a marked increase of transmembrane (tm) versus soluble (s) TNF in SpA versus RA together with a decrease in the enzymatic activity of ADAM17. In contrast with the destructive polysynovitis observed in classical TNF overexpression models, mice overexpressing tmTNF developed axial and peripheral joint disease with synovitis, enthesitis, and osteitis. Histological and radiological assessment evidenced marked endochondral new bone formation leading to joint ankylosis over time. SpA-like inflammation, but not osteoproliferation, was dependent on TNF-receptor I and mediated by stromal tmTNF overexpression. Collectively, these data indicate that TNF can drive distinct inflammatory pathologies. We propose that tmTNF is responsible for the key pathological features of SpA.

## Teaser

The authors show that SpA synovitis comprises less sTNF and more tmTNF expression compared to RA synovitis associated with less active ADAM17. Moreover, tmTNF overexpression induces SpA-like pathophysiology in mice. tmTNF signaling through TNF-RI induced inflammation, but not osteoproliferation.

## Introduction

Spondyloarthritis (SpA) is the second most frequent form of chronic inflammatory arthritis (Dougados and Baeten, 2011). This progressive and debilitating condition comprises several pathophysiologically related but clinically distinct phenotypes, including ankylosing spondylitis, psoriatic arthritis, inflammatory bowel disease–related SpA, reactive arthritis, and undifferentiated SpA (Baeten et al., 2013). All these subforms of SpA are characterized by inflammation of the spine and/or peripheral joints, sometimes in association with extra articular manifestations in the skin, gut, or eye. Moreover, SpA patients develop a specific phenotype of structural damage to the joints: whereas rheumatoid arthritis (RA) leads to erosive cartilage and bone damage without any new bone formation, axial and peripheral structural damage in SpA is characterized by a combination of joint tissue destruction (cartilage and bone) with marked presence of endochondral new bone formation (Lories et al., 2009; Vandooren et al., 2009). This formation of pathological new bone is pathognomonic for SpA and can ultimately lead to joint ankylosis.

Although the exact pathogenesis of SpA remains incompletely understood, ample evidence indicates a prominent role for the pro-inflammatory cytokine TNF in the pathology of this disease. Several single nucleotide polymorphisms in genes encoding molecules of the TNF pathway, such as *TNFRSF1A* (Davidson et al., 2011) and *TRADD* (Pointon et al., 2010), are associated with susceptibility to SpA. Moreover, therapeutic blockade of TNF by either mAbs or soluble decoy receptor constructs has a significant beneficial impact on signs and symptoms as well as pathology of the different SpA subforms (Baeten et al., 2001; Braun et al., 2002; Kruithof et al., 2006; Mease et al., 2000; Paramarta et al., 2013; Van Den Bosch et al., 2002). Surprisingly, however, experimental models of TNF overexpression do not accurately recapitulate the pathological features of SpA but rather phenocopy human RA (Keffer et al., 1991; Kollias et al., 2011; Kontoyiannis et al., 1999; Vieira-Sousa et al., 2015). Transgenic overexpression of human TNF results in spontaneous development of severe systemic inflammation and destructive RA-like polysynovitis (Keffer et al., 1991). Albeit blocking DKK-1, an inhibitor of the Wnt signaling pathway, can reverse the destructive phenotype into a remodeling phenotype characterized by new bone formation in synovial joints (Diarra et al., 2007), these mice still lack typical SpA features such as spondylitis and enthesitis. In a slightly different model, overexpression of mouse TNF by deletion of the adenylate-uridylate–rich elements in the murine TNF locus (*TNF^ΔARE^* mice) induces not only destructive polysynovitis, including sacroiliitis, but also inflammatory bowel disease (Armaka et al., 2008; Jacques et al., 2014; Kontoyiannis et al., 1999). The association with gut inflammation is of major interest when considering the clinical and genetic overlap between human SpA and Crohn’s disease and ulcerative colitis (De Wilde et al., 2015; Rudwaleit and Sieper, 2012), but also the *TNF^ΔARE^* model fails to recapitulate key pathological features of SpA such as spondylitis and endochondral new bone formation. The exact molecular and cellular mechanisms by which TNF contributes to SpA pathology thus remain poorly understood.

TNF is produced as a homo-trimeric transmembrane-bound cytokine; after enzymatic cleavage by ADAM17 (A disintegrin and metalloproteinase 17, also known as TNF converting enzyme [TACE]), the 17-kD soluble TNF is released extracellularly (Campbell et al., 2003). Both transmembrane (tm) and soluble (s) TNF are biologically active and can bind both TNF receptor I (p55) and TNF-RII (p75), albeit the literature about relative affinities of tmTNF and sTNF for each of the TNF receptors is still contradictory (Campbell et al., 2003; Faustman and Davis, 2010; Fischer et al., 2017; Grell et al., 1995). Our observation that sTNF levels in synovial fluid (SF) are significantly decreased in SpA versus RA despite similar levels of overall joint inflammation (Vandooren et al., 2009) urged us to revisit the role of the different TNF subforms in SpA.

## Results

### Elevated tmTNF expression in SpA synovitis

**Figure S1.**
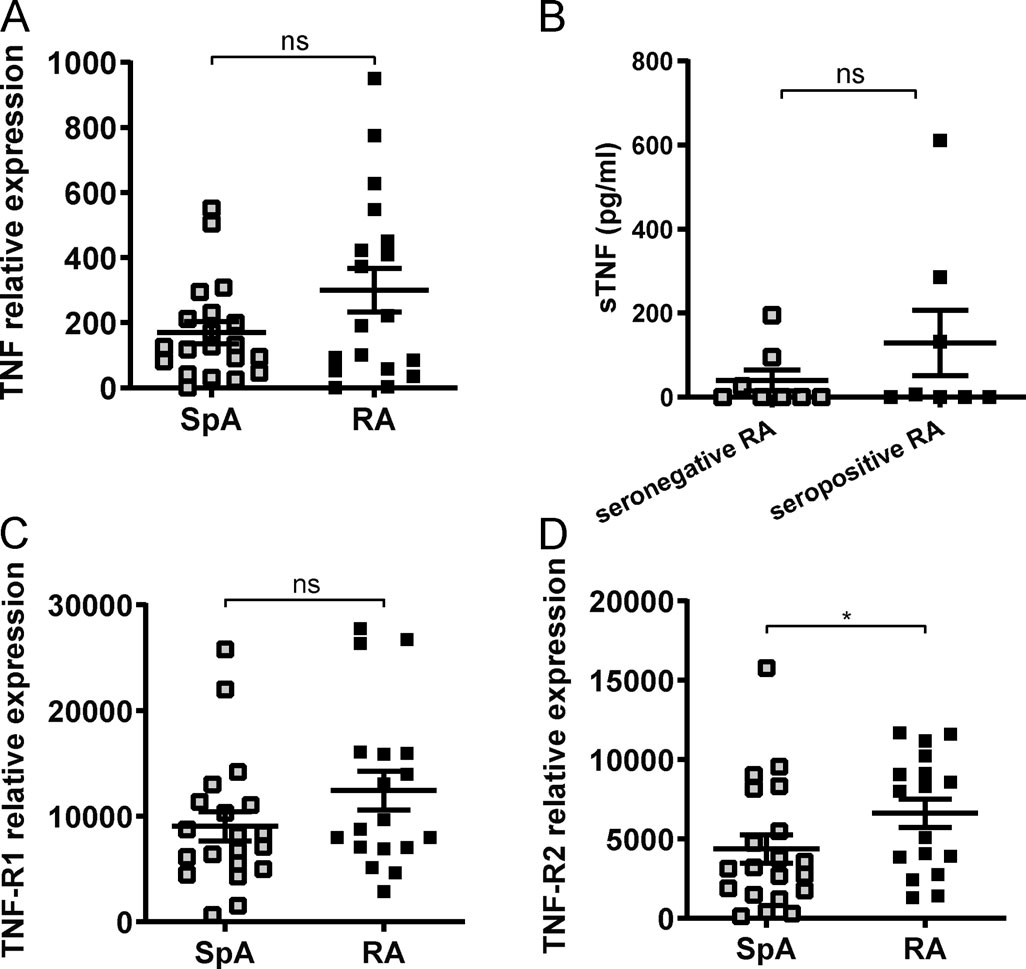
**Synovial TNF expression in SpA, seronegative, and seropositive RA patients. (A)** Relative TNF mRNA expression, measured by quantitative real-time PCR in synovial biopsies from SpA and RA patients (SpA, *n* = 20; RA, *n* = 17). **(B)** sTNF expression in SF from seronegative (*n* = 8) and seropositive (*n* = 8) RA, measured by ELISA. **(C and D)** Relative TNF-RI and TNF-RII mRNA expression, respectively, measured by quantitative real-time PCR in synovial biopsies from SpA and RA patients (SpA, *n* = 20; RA, *n* = 17). Normality was determined with a D’Agostino and Pearson omnibus test. The P value was determined by a Mann–Whitney test (A–D). Values depicted are means ± SEM; *, P < 0.05; ns, not significant. Data are representative of two independent experiments.

**Figure 1.**
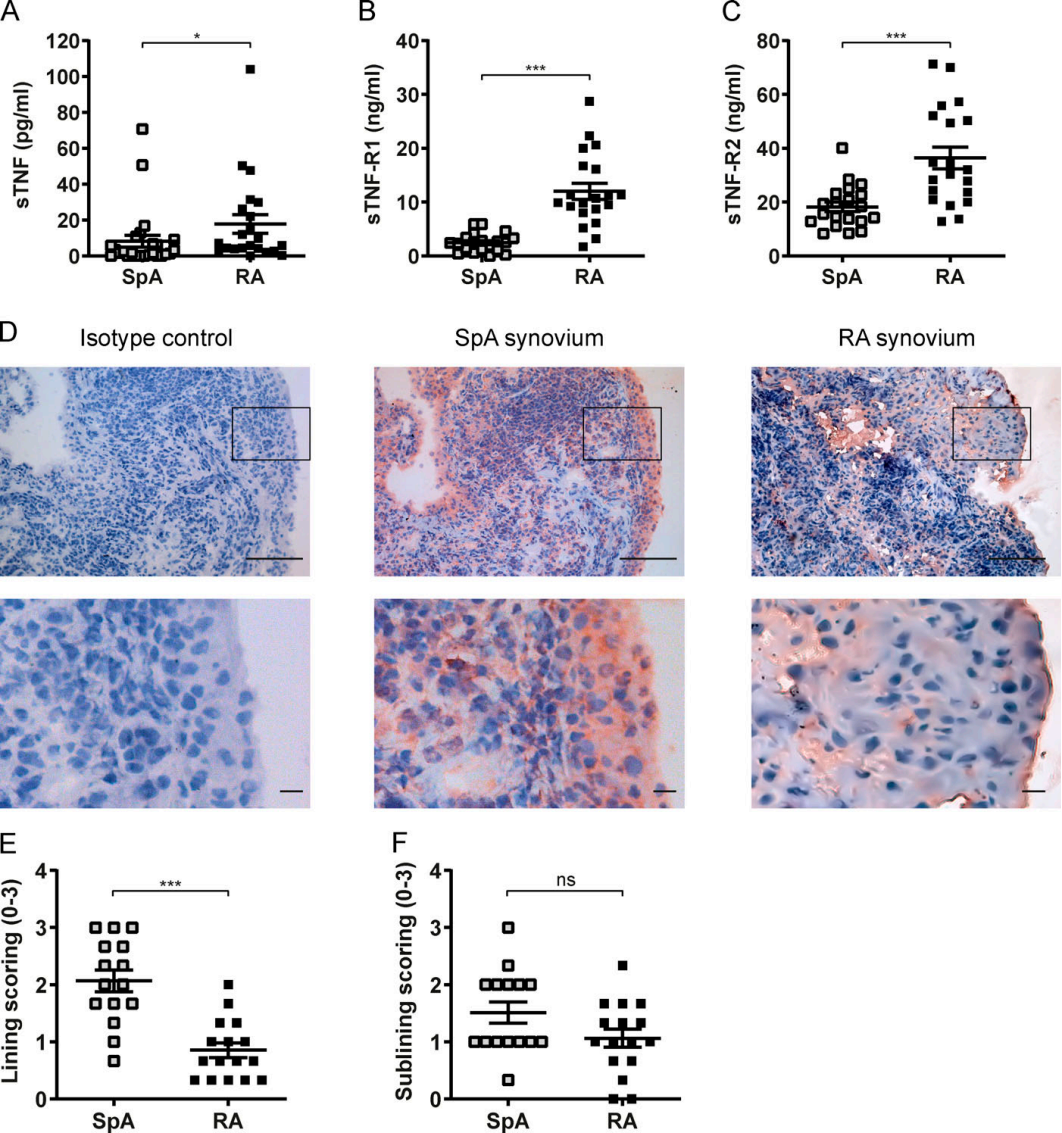
**TNF and TNF receptor expression in SpA versus RA synovitis. (A–C)** sTNF, TNF-RI, and TNF-RII expression in SF, respectively, measured by ELISA (SpA, *n* = 20; RA, *n* = 20). **(D)** TNF protein expression measured by immunohistochemistry in synovial biopsies from SpA and RA patients. Lower panels are magnified view of box. Scale bars, 100 µm (top), 10 µm (bottom). **(E and F)** Semi-quantitative score (0–3) of, respectively, the TNF expression in the lining layer and sublining (SpA, *n* = 15; RA, *n* = 16). The P value was determined by a Mann–Whitney test (A, C, E, and F) or an unpaired *t* test (B). Data are representative of two independent experiments with two replicates (A–C) per sample. Values depicted are means ± SEM; *, P < 0.05; ***, P < 0.001; ns, not significant.

We previously demonstrated that SF sTNF levels were significantly lower in peripheral SpA than RA despite similar degrees of overall inflammation (Vandooren et al., 2009) and good responsiveness of peripheral SpA to anti-TNF treatment (Paramarta et al., 2013). To explore this paradoxical finding, we first assessed in more detail the TNF expression in SpA synovitis using RA as control. Quantitative PCR (qPCR) analysis of synovial tissue biopsies revealed similar TNF mRNA levels in both diseases (Fig. S1 A). Confirming our previous data (Vandooren et al., 2009), SF levels of sTNF protein were significantly lower in SpA (median: 1.77 pg/ml) versus RA (median: 6.52 pg/ml; P = 0.012; Fig. 1 A). This difference between both diseases was not related to specific subgroups such as seronegative versus seropositive RA (Fig. S1 B). The low levels of sTNF in SpA SF were also not related to an increase in the decoy receptors capturing sTNF as the soluble TNF receptor I (p55; SpA: median 2.45 ng/ml versus RA: median 10.3 ng/ml; P < 0.001) and receptor II (p75; SpA: median 17 ng/ml versus RA: median 31.15 ng/ml; P < 0.001) were also significantly decreased in SpA versus RA SF (Fig. 1, B and C), despite comparable synovial tissue mRNA levels in both conditions (Fig. S1, C and D). As the 17 kD sTNF as well as sTNF-RI and sTNF-RII are generated by enzymatic cleavage by ADAM17, we explored if the decrease in sTNF in SpA SF could be related to altered cleavage and a disturbed balance between sTNF and the 26 kD tmTNF expressed on the surface of various cell subsets (Campbell et al., 2003). Using an anti-TNF antibody that does not bind to TNF captured by either one of the TNF receptors expressed on the cell surface (Gerspach et al., 2000), immunohistochemical analysis revealed significantly increased cellular staining for TNF in the synovial lining layer, but not the synovial sublining layer, of SpA versus RA (SpA: median 2 versus RA: median 0.667; P < 0.001; Fig. 1, D–F), indicating increased tmTNF expression. Collectively, these data demonstrate a relative overexpression of tmTNF over sTNF in SpA versus RA synovitis.

### Decreased ADAM17 activity in SpA

**Figure S2.**
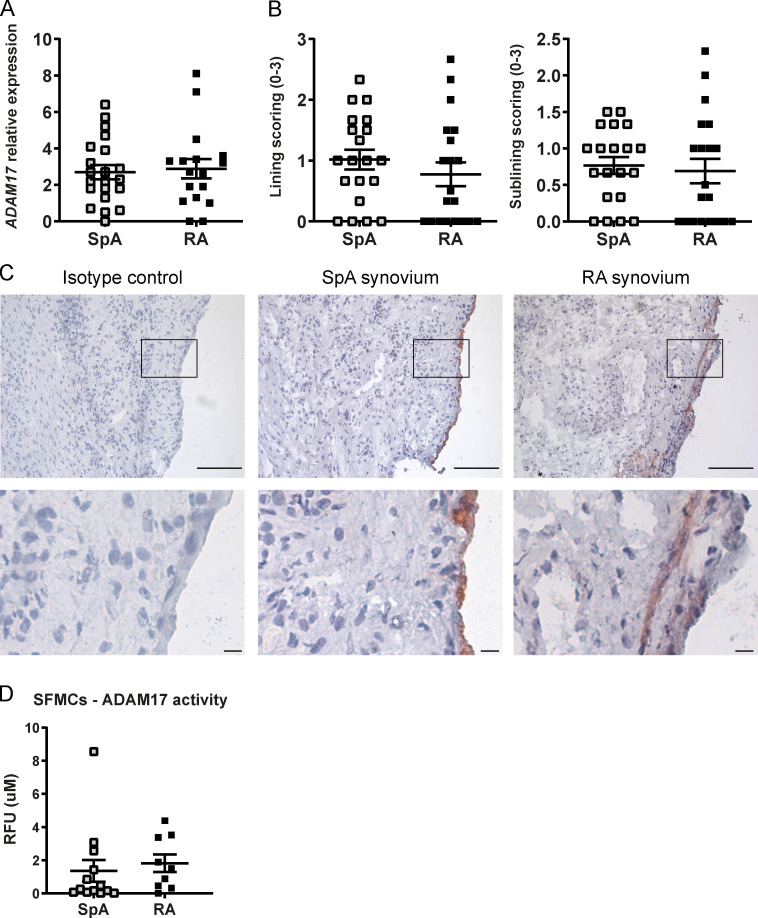
**Decreased ADAM17 activity in SpA SFMCs and PBMCs compared with RA SFMCs and PBMCs. (A)** Relative *ADAM17* mRNA expression measured by quantitative real-time PCR in synovial biopsies from SpA and RA patients (SpA, *n* = 21; RA, *n* = 17). **(B)** Semi-quantitative score (0–3) of the ADAM17 protein expression in the synovial lining layer and sublining, respectively, as assessed by immunohistochemistry (SpA, *n* = 20; RA, *n* = 20). **(C)** ADAM17 protein expression measured by immunohistochemistry in synovial biopsies from SpA and RA patients. Lower panels are magnified view of box. Scale bars, 100 µm (top), 10 µm (bottom). **(D)** Enzymatic activity of ADAM17 in SFMCs of SpA (*n* = 13) and RA (*n* = 9) patients. Normality was determined with a D’Agostino and Pearson omnibus test. The P value was determined by a Mann–Whitney test (A and D) or an unpaired *t* test (B). Values depicted are means ± SEM. Data are representative of two independent experiments (A, B, D, and E). RFU, relative fluorescence units.

**Figure 2.**
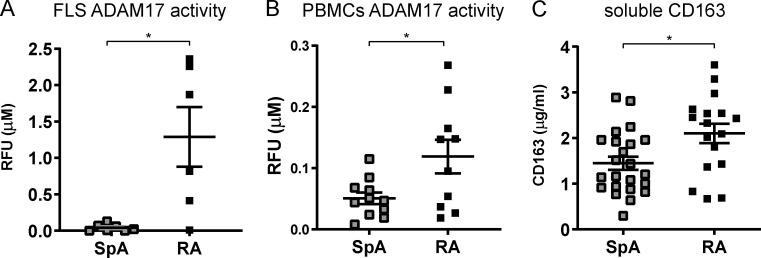
**Decreased enzymatic activity of ADAM17 in SpA FLS and PBMCs. (A)** Enzymatic activity of ADAM17 in total cell lysates of FLS cultures (SpA FLS *n* = 7; RA FLS *n* = 6). **(B)** Enzymatic activity of ADAM17 in PBMCs from SpA (*n* = 11) and RA (*n* = 10) patients. **(C)** Soluble CD163 expression in SF measured by ELISA (SpA, *n* = 23; RA, *n* = 17). The P value was determined by a Mann–Whitney test (A) or an unpaired *t* test (B and C). Data are representative of two independent experiments with two replicates (A, B, and C) per sample. Values depicted are means ± SEM; *, P < 0.05. RFU, relative fluorescence units.

To address the question why an altered balance in tmTNF versus sTNF expression in SpA synovitis compared with RA synovitis is observed, we investigated the expression and activity of ADAM17. ADAM17 (or TACE) is the only disintegrin and metalloproteinase capable of cleaving TNF. qPCR analysis of synovial tissue biopsies revealed similar *ADAM17* mRNA levels in both diseases (Fig. S2 A). Also, protein expression by immunohistochemical analyses revealed similar expression of *ADAM17* in SpA and RA synovial tissue (Fig. S2, B and C). Next, we analyzed the enzymatic activity of ADAM17 in SpA and RA fibroblast-like synoviocyte (FLS) cultures. ADAM17 was less active in SpA FLS compared with RA FLS (SpA: median 0.021 versus RA: median 1.3437; P = 0.0487; Fig. 2 A). There was also a significant decrease in ADAM17 activity in SpA peripheral blood mononuclear cells (PBMCs) compared with RA PBMCs, indicating that the relative difference in ADAM17 activity in the SpA versus RA joint is related to a systemic impairment of ADAM17 activity in SpA (Fig. 2 B). The same trend toward decreased ADAM17 activity was observed in SpA SF-derived monocytes (SFMCs) compared with RA SFMCs (Fig. S2 D). To investigate whether ADAM17 enzymatic activity is decreased not only ex vivo in SpA-derived cells but also in vivo in SpA synovitis, we measured the levels of soluble CD163 in SF. CD163 is a scavenger receptor expressed by macrophages and exclusively cleaved by ADAM17 (Etzerodt et al., 2010). We previously demonstrated a marked and consistent increase in CD163 membrane staining in SpA versus RA synovitis (Ambarus et al., 2012; Baeten et al., 2000, 2002, 2004b, 2005; Vandooren et al., 2009). In line with the in vitro demonstration of decreased ADAM17 activity, we demonstrated that sCD163 levels in SF were decreased in SpA versus RA synovitis (Fig. 2 C). Together with the lower levels of soluble TNF-RI and TNF-RII, two other substrates of ADAM17, these results indicate that the altered balance in tmTNF versus sTNF in SpA is related to a decrease in enzymatic activity of ADAM17.

### tmTNF-overexpressing mice develop a SpA phenotype

**Figure 3.**
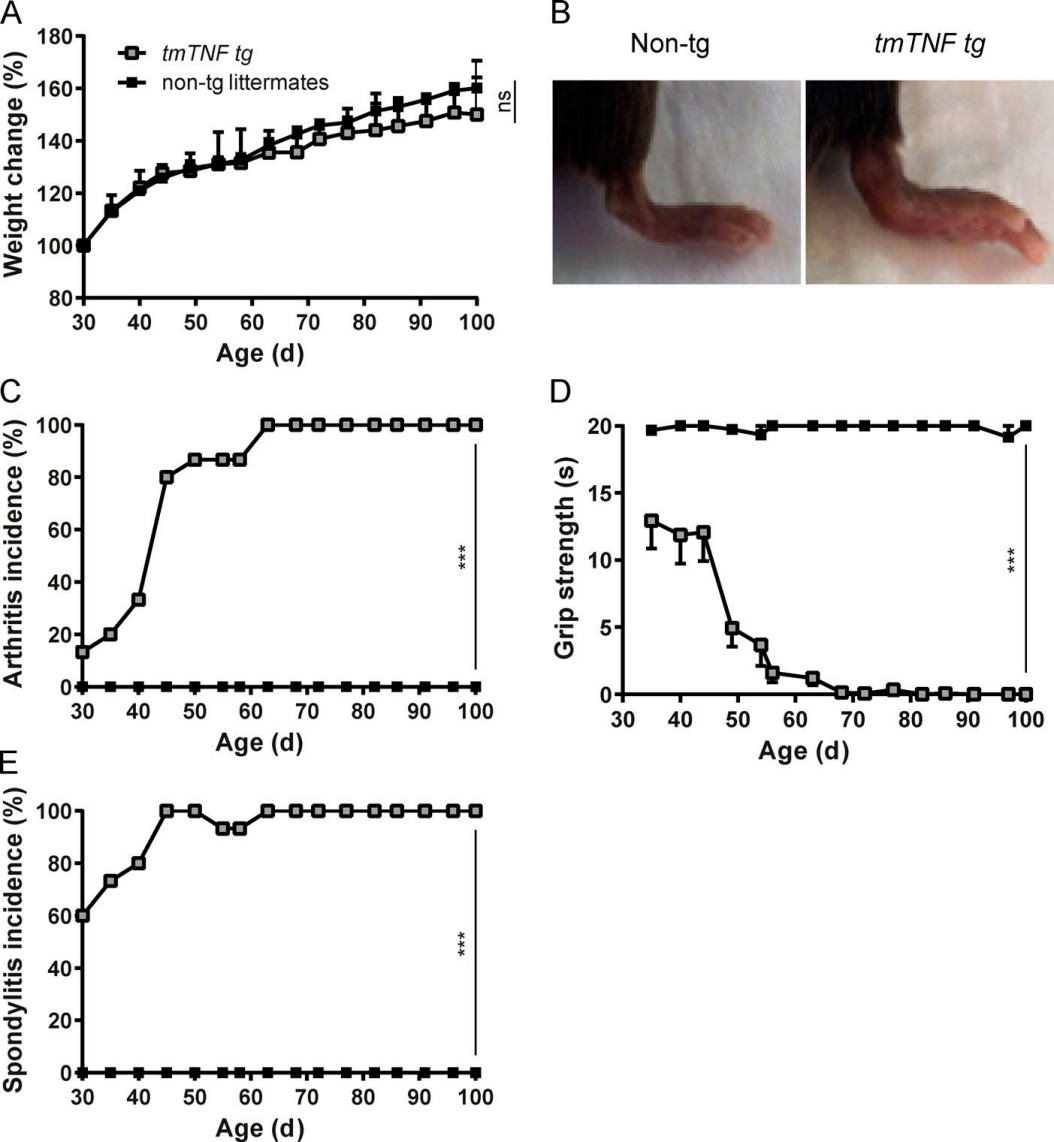
**tmTNF-overexpressing mice develop clinical symptoms of arthritis and spondylitis. (A)** Percentage of weight gain compared with day 30 in *tmTNF tg* mice and nontg littermates. **(B)** Picture of 100-d-old nontg littermate and *tmTNF tg* mice, the latter displaying slight swelling of hind paws. **(C)** Arthritis incidence over time. **(D)** Grip strength in seconds over time is depicted (maximal 20 s of grip strength were measured per time point). **(E)** Spondylitis incidence over time, P < 0.001 for every time point. Weight curves were analyzed with a two-way ANOVA analysis with Sidak’s multiple comparisons post-test (A). Area under the curve difference was determined with a Mann–Whitney test (A). A log-rank (Mantel–Cox) test was performed for arthritis incidence (C). A Fisher’s exact test was performed for spondylitis incidence for each time point (E). Data are representative of four independent experiments with at least six mice per group. Values are mean ± SEM; *n* = 15 per group; ***, P < 0.001; ns, not significant.

**Figure S3.**
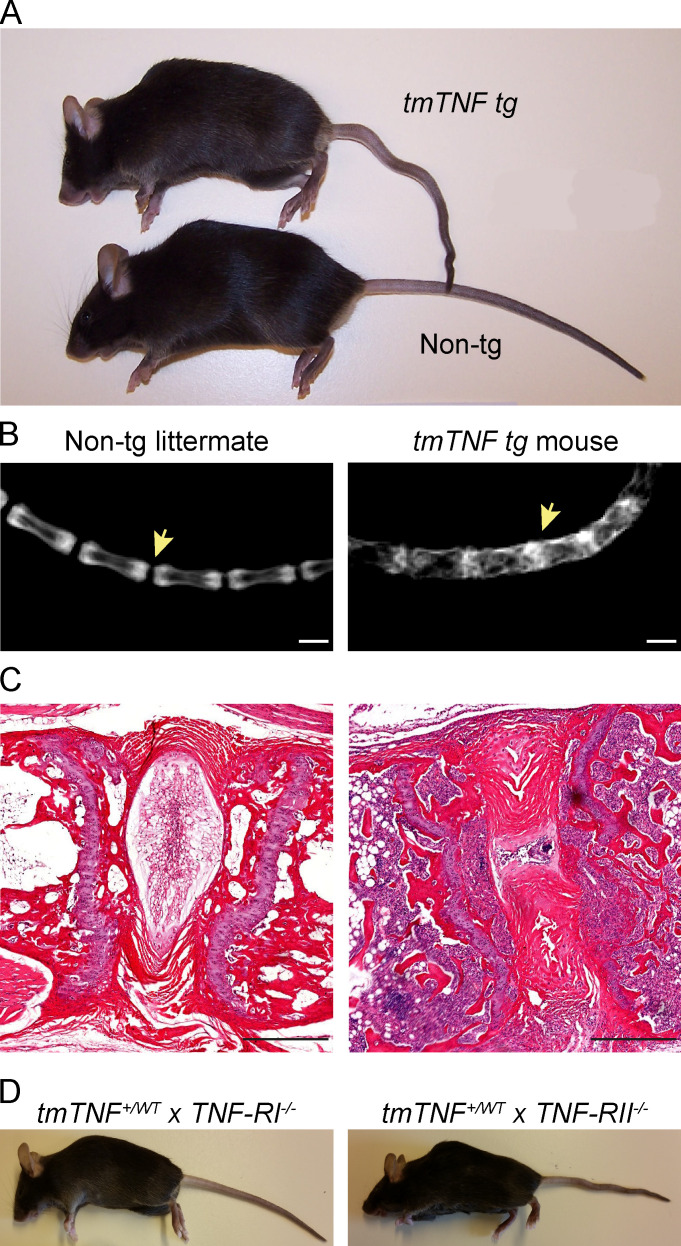
**Joint fusion of vertebrae in *tmTNF tg* mice. (A)** Picture of 100-d-old *tmTNF tg* mice and a nontg littermate revealing hunchback formation and a crinkled tail in the *tmTNF tg* mouse, which is absent in the nontg littermate control. **(B)** Radiographical images of the tail in 8-mo-old nontg and *tmTNF tg* mouse. Arrow indicates spinal fusion in the *tmTNF tg* tail, while there is intervertebral space in the nontg mouse. Scale bars, 1 mm. **(C)** H&E staining of an 8-mo-old nontg littermate and *tmTNF tg* mouse showing ankylosis in the *tmTNF tg* tail and destruction of the intervertebral disc. Scale bars, 200 µm. **(D)** Photo of a *tmTNF^+/WT^xTNF-RI^−/−^* mouse without clinical symptoms and a photo of a *tmTNF^+/WT^xTNF-RII^−/−^* mouse with hunchback formation and a crinkled tail. *n* = 3 mice per group in two independent experiments.

To explore the potential functional role of tmTNF in SpA pathogenesis, we studied the phenotype of the *TgA86* mouse. This mouse overexpresses systemically a mutant murine *TNF* gene with a defect in the cleavage site for ADAM17 (muTNF_Δ1-12_), leading to a specific systemic increase of tmTNF but not sTNF expression (Alexopoulou et al., 1997). In contrast to other mouse models of TNF overexpression (Keffer et al., 1991; Kontoyiannis et al., 1999), the *tmTNF* transgenic (*tmTNF tg*) mice did not show signs of systemic inflammation such as weight loss or growth defects over a 100-d follow-up period (Fig. 3 A). In line with previous reports (Alexopoulou et al., 1997; Edwards et al., 2006), the mice did, however, develop swelling and deformation of the front and hind paws (Fig. 3 B), starting already at 30 d of age and reaching a 100% incidence at day 63 (Fig. 3 C). This peripheral arthritis was associated with a rapid and profound loss of grip strength (Fig. 3 D). Additionally, the *tmTNF tg* mice developed clinical spondylitis, characterized by the development of a hunchback and a crinkled tail (Fig. S3 A), starting at 25 d of age and reaching a 100% incidence at day 45 (Fig. 3 E). No arthritis or spondylitis was observed in the WT (nontg) littermate controls.

**Figure 4.**
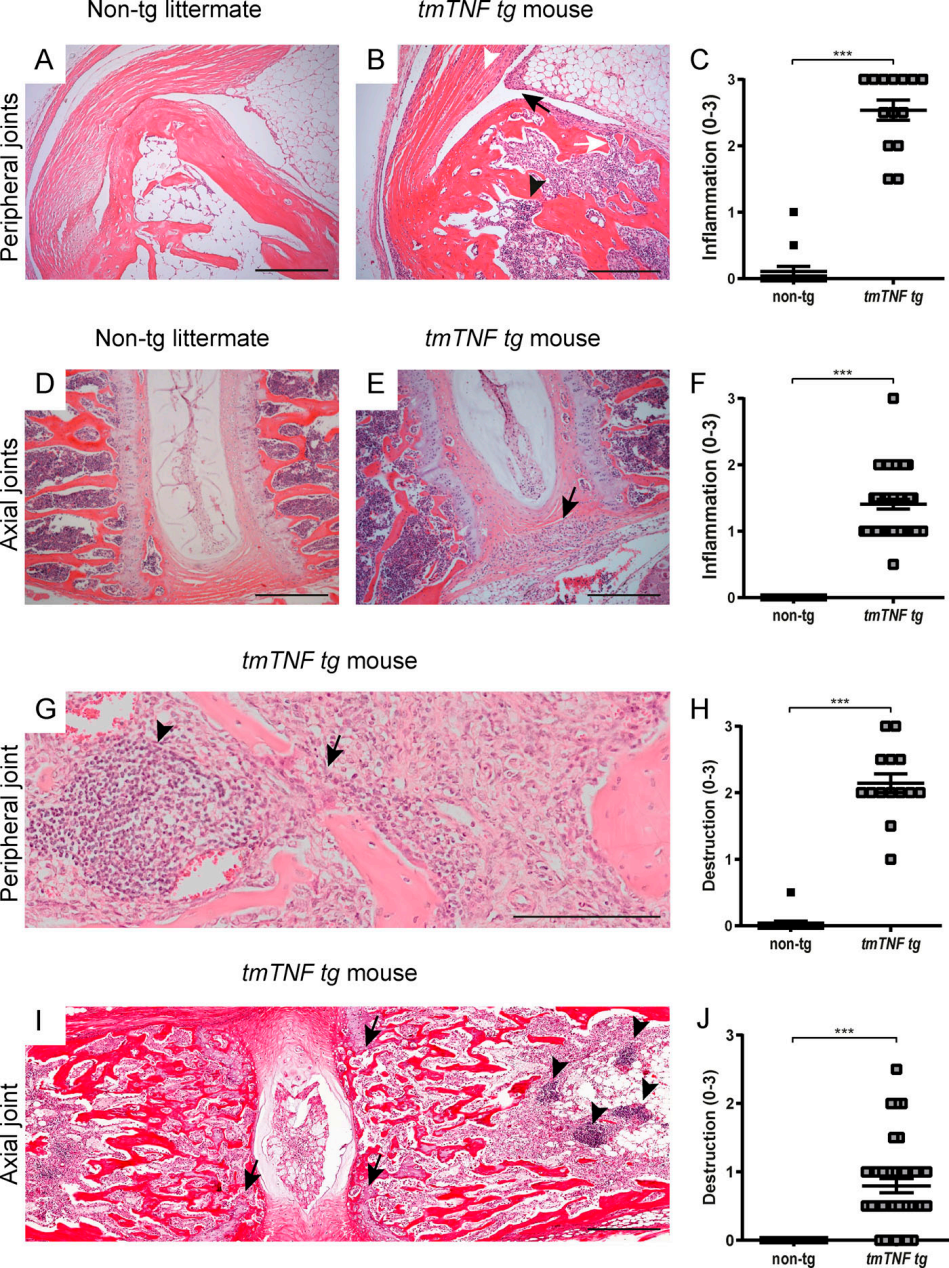
**Inflammation and bone and cartilage destruction in peripheral and axial joints of *tmTNF tg* mice. (A)** H&E staining of ankle joint of a 100-d-old nontg littermate. **(B)** H&E staining of a 100-d-old *tmTNF tg* mouse reveals synovitis (black arrow), osteitis (white arrow), enthesitis (white arrowhead), and lymphoid aggregates (black arrowhead). Scale bars, 200 µm. **(C)** Semi-quantitative score (0–3) of inflammation in ankle joints of *tmTNF tg* mice compared with nontg littermates (nontg, *n* = 7; *tmTNF tg*, *n* = 7, both ankles per mouse, in two independent experiments). **(D)** H&E staining of an axial joint of a 100-d-old nontg littermate. **(E)** H&E staining of axial joint sections of a 100-d-old *tmTNF tg* mouse, displaying cellular infiltrate (black arrow) in the connective tissue next to the intervertebral disc. Scale bars, 200 µm. **(F)** Semi-quantitative score (0–3) of inflammation in axial joints of *tmTNF tg* mice compared with nontg littermates (nontg *n* = 30, *tmTNF tg* *n* = 44, in four independent experiments). **(G)** H&E staining of an ankle of a 100-d-old *tmTNF tg* mouse with destruction (black arrow) and lymphoid aggregate (black arrowhead). Scale bars, 100 µm. **(H)** Semi-quantitative score (0–3) of destruction in peripheral joints of *tmTNF tg* mice compared with nontg littermates (nontg, *n* = 7; *tmTNF tg*, *n* = 7, both ankles per mouse, in two independent experiments). **(I)** H&E staining of an axial joint of a 100-d-old *tmTNF tg* mouse indicating destruction by disruption of the cartilage endplate (black arrows) and lymphoid aggregate (black arrowheads). Scale bars, 200 µm. **(J)** Semi-quantitative score (0–3) of destruction in axial joints of *tmTNF tg* mice compared with nontg littermates (nontg, *n* = 29; *tmTNF tg*, *n* = 37, in four independent experiments). The P value was determined by a Mann–Whitney test (C, F, H, and J). Data are representative of two independent experiments with two replicates (A–F) per sample. Values are mean ± SEM; ***, P < 0.001.

**Figure S4.**
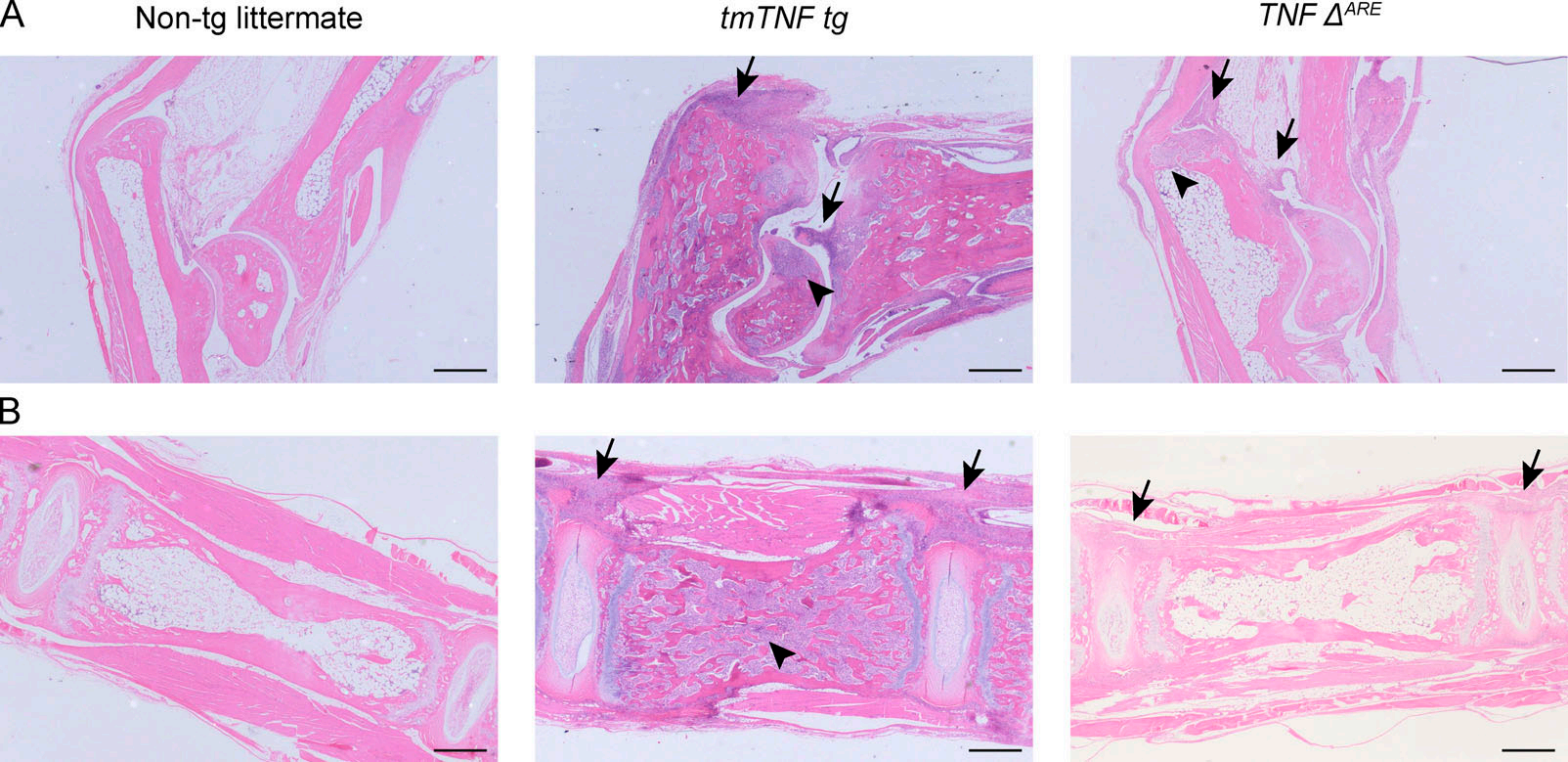
**Comparison of inflammation in *tmTNF tg* and *TNF^ΔARE^* mice axial and peripheral joints**. **(A)** H&E stainings of ankle joints of a nontg (*tmTNF tg* littermate), *tmTNF tg*, and *TNF^ΔARE^* mouse reveals that both *tmTNF tg* and *TNF^ΔARE^* mice have inflammation and destruction (arrowhead). In the *tmTNF tg* mouse, the inflammation is along the Achilles tendon and in the synovium (arrow), whereas the inflammation in the *TNF^ΔARE^* mouse is mostly limited to the synovium (arrow). **(B)** H&E stainings of axial joints of a nontg, *tmTNF tg*, and *TNF^ΔARE^*. The *tmTNF tg* axial joint reveals extensive inflammation along the ligaments (arrow) and in the bone marrow (arrowhead), while the *TNF^ΔARE^* mouse shows minimal inflammation along the ligaments without osteitis. *n* = 3 mice per group. Scale bars, 500 µm.

To confirm these clinical findings and investigate the pathological processes underlying the arthritic and spondylitic phenotype of the *tmTNF tg* mice, we assessed histologically ankles and spinal (lumbar spine and tail) sections from *tmTNF tg* mice and their age- and gender-matched nontg littermates at 100 d of age. None of the nontransgenic littermates had synovitis, enthesitis and osteitis of the peripheral joints (Fig. 4 A), whereas all *tmTNF tg* mice depicted a marked leukocytic infiltration of the synovial tissue, the enthesis, and the bone marrow (Fig. 4, B and C; P < 0.001). Likewise, the axial skeleton of the nontg littermates had no axial pathology (Fig. 4 D), whereas the *tmTNF tg* mice showed an inflammatory infiltration along the ligaments in connective tissue at the border of the intervertebral discs (Fig. 4 E) as well as lymphoid aggregates in the bone marrow (Fig. 4, F and I; P < 0.001). In line with the commonly used TNF-overexpressing mice such as the *hTNF* mice (*Tg197*) and *TNF^ΔARE^* mice where peripheral joint inflammation is associated with focal cartilage and bone destruction (Keffer et al., 1991; Kontoyiannis et al., 1999), histological analysis revealed clear, albeit relatively mild, focal bone destruction of the peripheral joints (Fig. 4, G and H; P < 0.001). With respect to peripheral arthritis, the *tmTNF tg* mice also had extensive enthesitis with adjacent synovitis and osteitis, whereas the *TNF^ΔARE^* mice only had clear synovitis without widespread enthesitis or osteitis (Fig. S4 A). Also, the spine of *tmTNF tg* mice showed clear signs of focal destruction, albeit mild and merely restricted to disruption of the cartilage endplate (Fig. 4, I and J; P < 0.001). For spondylitis, *TNF^ΔARE^* mice showed minimal inflammatory cell infiltration along the ligaments of the spine without signs of structural damage, whereas *tmTNF tg* mice had massive inflammation along the ligaments as well as in the bone marrow (Fig. S4 B).

Extensive histological analysis of extra articular sites revealed no inflammation or other pathology in eyes, skin, colon, and intestine of 100-d-old *tmTNF tg* mice (data not shown). Taken together, these data indicate that selective overexpression of tmTNF leads to axial and peripheral joint pathology reminiscent of human SpA (synovitis, enthesitis, and osteitis) in the absence of severe systemic disease and/or extra articular manifestations.

### tmTNF drives osteoproliferative joint remodeling

**Figure S5.**
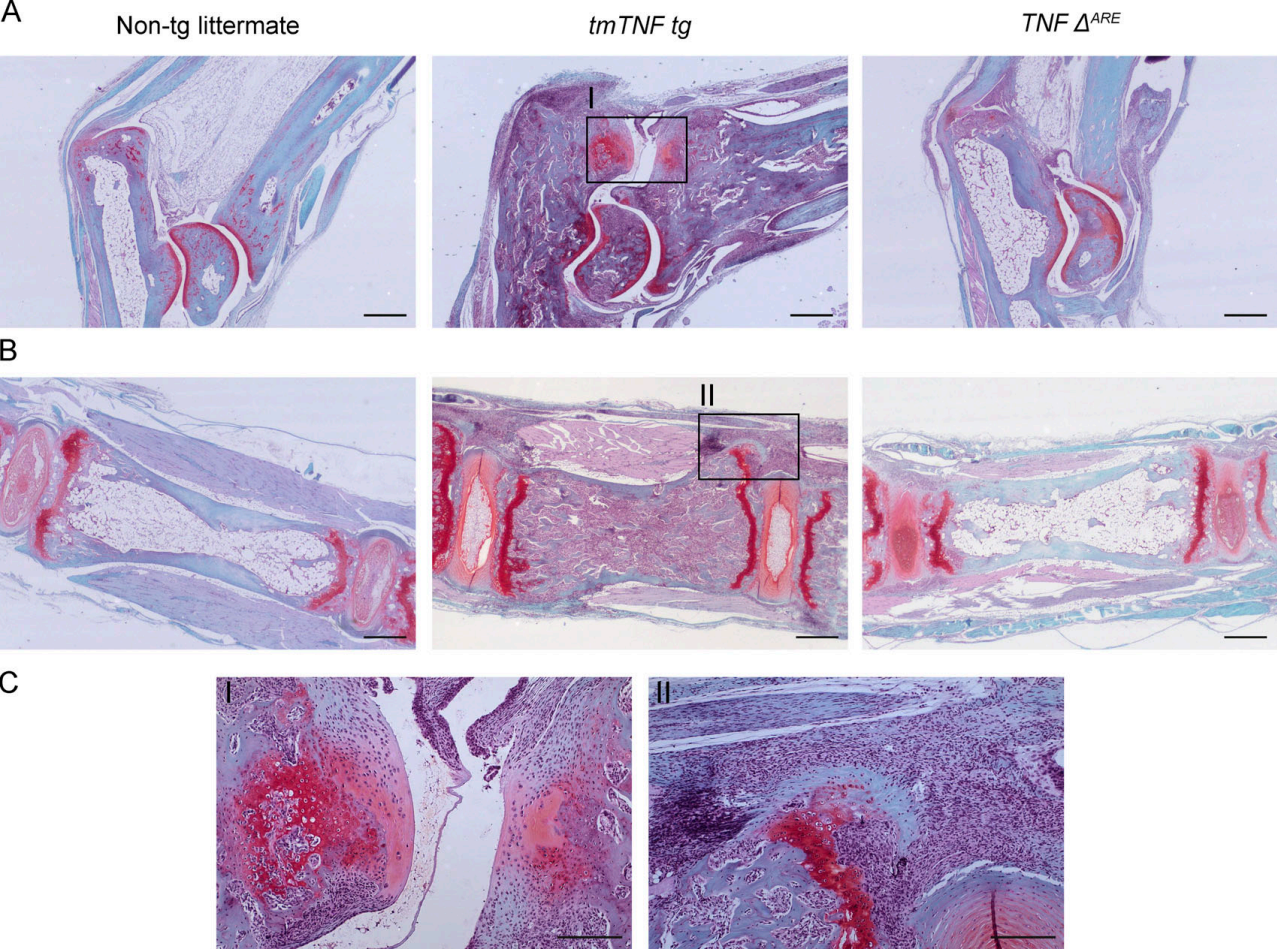
**Endochondral new bone formation is only present in *tmTNF tg* mouse. (A)** Safranin-O/fast green stainings of ankle joints of a nontg, *tmTNF tg*, and *TNF^ΔARE^* mouse reveals that the *tmTNF tg* ankle has clear bone formation, while the *TNF^ΔARE^* and nontg do not. Scale bars, 500 µm. **(B)** Safranin-O/fast green stainings of axial joints of a nontg, *tmTNF tg*, and *TNF^ΔARE^* mouse. The *tmTNF tg* axial joint reveals inflammation adjacent to bone formation, whereas the *TNF^ΔARE^* and nontg do not have these features. Scale bars, 500 µm. **(C)** Magnified view of *tmTNF tg* axial and ankle joint of region with endochondral ossification. Scale bars, 200 µm. *n* = 3 mice per group.

**Figure 5.**
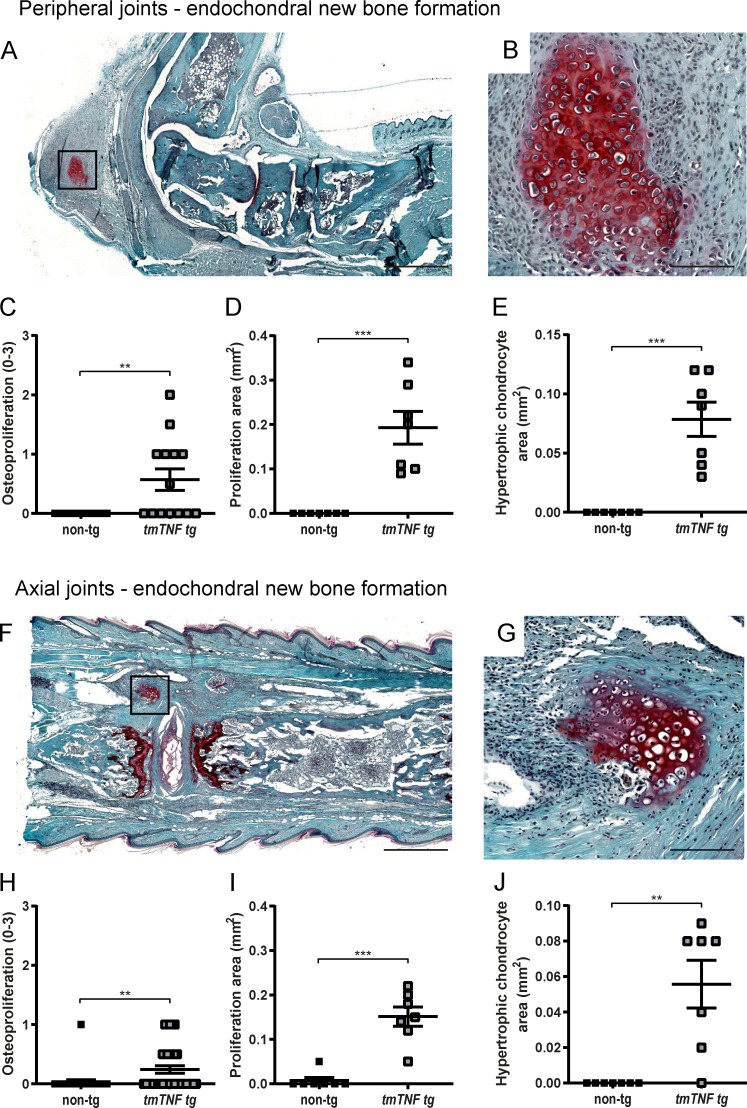
**Osteoproliferation and endochondral new bone formation in *tmTNF tg* mice. (A and B)** Safranin-O/fast green staining to detect proteoglycans (square in A) in an ankle joint of a *tmTNF tg* mouse. Scale bars, 500 µm (A) and 50 µm (B). **(C)** Semi-quantitative score (0–3) of endochondral new bone formation in ankle joints of *tmTNF tg* mice compared with nontg littermates (nontg, *n* = 7; *tmTNF tg*, *n* = 7, both ankles per mouse, in two independent experiments). **(D and E)** Histomorphometric analyses: proliferation area and the hypertrophic chondrocyte area within this proliferation area (nontg, *n* = 7; *tmTNF tg*, *n* = 7, in two independent experiments). **(F–J)** Safranin-O/fast green staining to detect proteoglycans (square in F) in an axial joint of a *tmTNF tg* mouse. Scale bars, 500 µm (F) and 50 µm (G). **(H)** Semi-quantitative score (0–3) of endochondral new bone formation in axial joints of *tmTNF tg* mice compared with nontg littermates (nontg, *n* = 28; *tmTNF tg*, *n* = 35, in four independent experiments). **(I and J)** Histomorphometric analyses: proliferation area and the hypertrophic chondrocyte area within this proliferation area (nontg, *n* = 7; *tmTNF tg*, *n* = 7, in two independent experiments). The P value was determined by a Mann–Whitney test (C–E and H–J). Values are mean ± SEM; **, P < 0.01; ***, P < 0.001.

**Figure 6.**
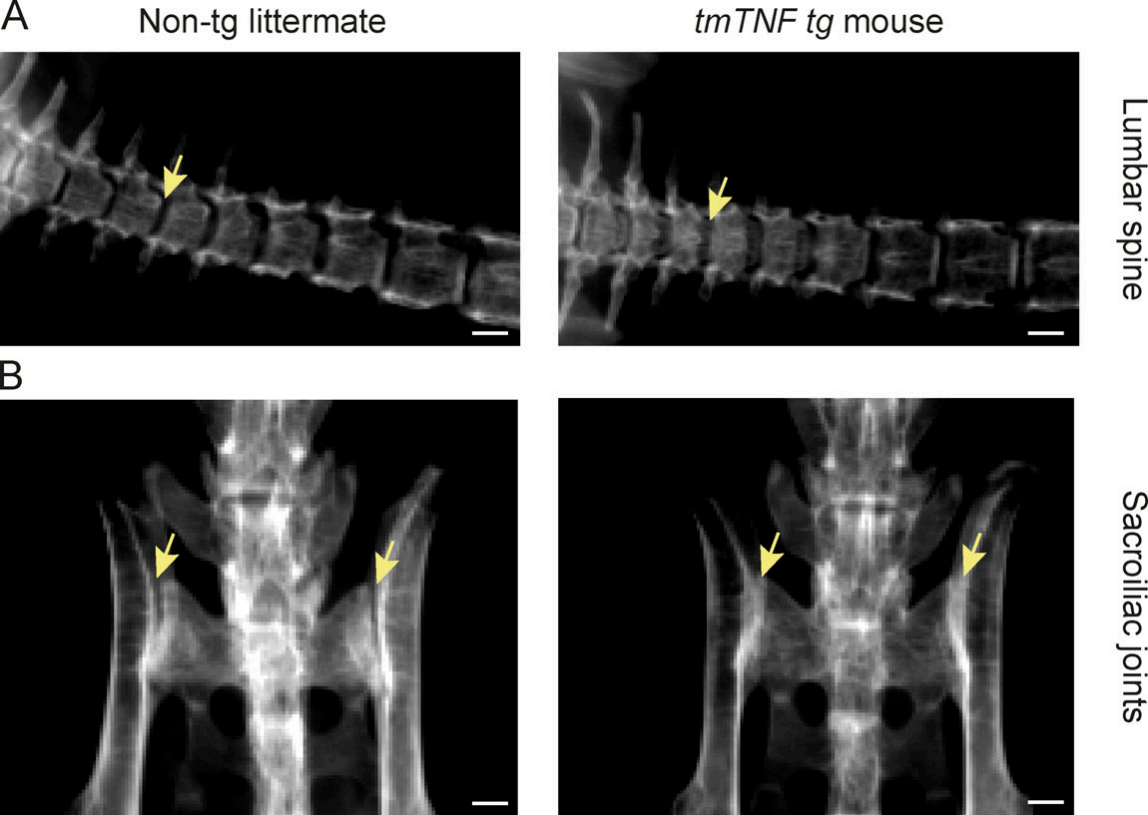
**Joint fusion of vertebra and sacroiliitis in 8-mo-old *tmTNF tg* mice. (A and B)** Radiographical images of an 8-mo-old nontg littermate and a *tmTNF tg* mouse. **(A)** Radiographical images of the lumbar spine. Arrow indicates site of joint margin blurring in the *tmTNF tg* mice, while blurring is absent in nontg littermates. Scale bars, 1 mm. **(B)** Radiographical images of the sacroiliac joints. Arrows indicate joint margin blurring in the *tmTNF tg* mouse, which is absent in the nontg littermate. Scale bars, 1 mm. *n* = 3 mice per group.

In *hTNF* mice (*Tg197*) and *TNF^ΔARE^* mice, the inflammatory polysynovitis is characterized by a profound focal degradation of cartilage and bone in the absence of significant new bone formation, recapitulating the structural phenotype of human RA (Keffer et al., 1991; Kontoyiannis et al., 1999). In sharp contrast, however, the *tmTNF tg* mice displayed clear features of new bone formation in the inflamed peripheral joints (Fig. S5 A). Safranin-O staining of proteoglycans revealed peri-articular chondro-proliferative lesions in the ankles of *tmTNF tg* mice but not in nontg littermates (Fig. 5, A–C; P = 0.0058). This was confirmed by histomorphometric analyses of the complete osteophyte proliferation area (Fig. 5 D; P < 0.001) as well as the specific hypertrophic chondrocyte area (Fig. 5 E; P < 0.001). Similarly for the axial skeleton, hypertrophic chondrocytes were seen in the connective tissue at the edge of the intervertebral disc of the axial skeleton in the *tmTNF tg* animals but not in their nontg littermates (Fig. 5, F–H; P = 0.004), which was again confirmed by histomorphometric analyses of the complete osteophyte proliferation area (Fig. 5 I; P = 0.001) as well as the specific hypertrophic chondrocyte area (Fig. 5 J; P = 0.005). To assess if these peri-articular chondro-proliferative lesions lead to endochondral new bone formation, 8-mo-old *tmTNF tg* and nontg littermate animals were subjected to radiographical analysis. Whereas no new bone formation was observed in the nontg controls (Fig. 6, A and B), the *tmTNF tg* mice displayed obvious vertebral fusion in the absence of pronounced bone erosions in the lumbar spine (Fig. 6 A) and the tail (Fig. S3 B). By histological analyses, we confirmed the vertebral fusion in these 8-mo-old *tmTNF tg* mice (Fig. S3 C). Moreover, these radiographical images revealed blurring of the joint space and ankylosis of the sacroiliac joints in the *tmTNF tg* animals (Fig. 6 C). Collectively, the histological and radiological analysis concord to indicate that the clinical SpA induced by selective tmTNF overexpression is associated with a remodeling, rather than destructive, structural phenotype reminiscent of human SpA.

### tmTNF-induced SpA-like inflammation requires TNF-RI signaling

**Figure 7.**
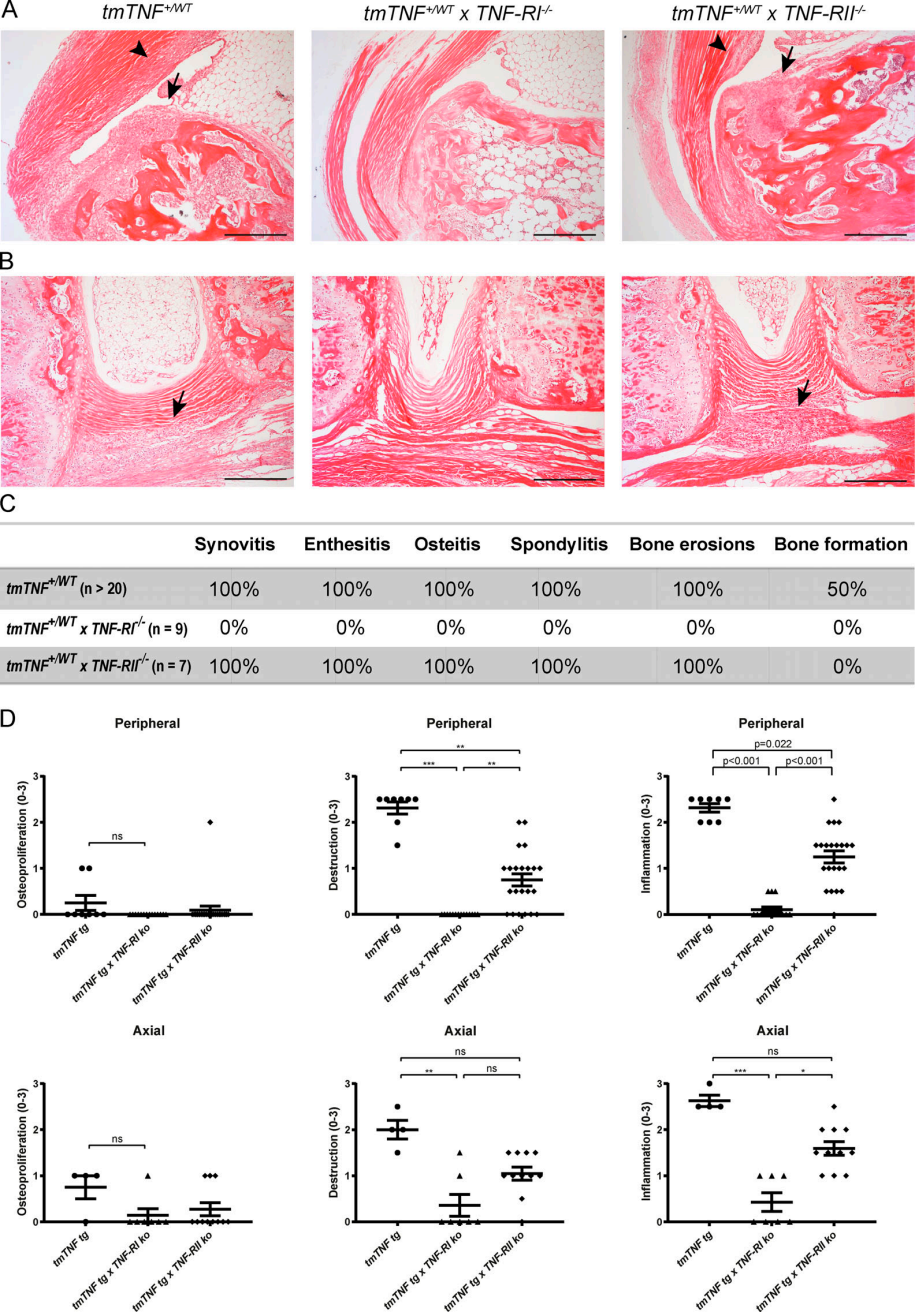
**tmTNF-induced SpA-like inflammation requires TNF-RI receptor signaling. (A)** H&E stainings of an ankle joint of a 100-d-old *tmTNF^+/WT^ tg* mouse, depicting inflammation and destruction; an ankle joint of a 100-d-old *tmTNF^+/WT^xTNF-RI^−/−^* mouse without cellular infiltrate and an ankle joint of a 100-d-old *tmTNF^+/WT^xTNF-RII^−/−^* mouse. Inflammation is observed in the bone (osteitis, arrow) as well as in the enthesis (enthesitis, arrowhead). Scale bars, 200 µm. **(B)** H&E stainings of an axial joint of a *tmTNF^+/WT^ tg* mouse, depicting cellular infiltrate at the border of the intervertebral disc in the connective tissue; a axial joint of a *tmTNF^+/WT^xTNF-RI^−/−^* mouse without cellular infiltrate and a axial joint of a *tmTNF^+/WT^xTNF-RII^−/−^* mouse. Again, inflammation is observed at the edge of the intervertebral disc in the connective tissue (arrow). Scale bars, 200 µm. **(C)** Overview of pathological features of SpA observed in the three lines. *tmTNF^+/WT^ tg*: *n* > 20; old *tmTNF^+/WT^xTNF-RI^−/−^*: *n* = 9; *tmTNF^+/WT^xTNF-RII^−/−^*: *n* = 7. **(D)** Histological scoring of *tmTNF^+/WT^ tg*: *n* = 4; old *tmTNF^+/WT^xTNF-RI^−/−^*: *n* = 7; *tmTNF^+/WT^xTNF-RII^−/−^*: *n* = 11. Both ankles per mouse were assessed. The P value was determined by a Kruskal–Wallis test with Dunn’s multiple comparisons test (D). Data are representative of two independent experiments (A and B); C and D are two independent experiments. Values are mean ± SEM; *, P < 0.05; **, P < 0.01; ***, P < 0.001; ns, not significant.

To delineate the cellular and molecular mechanisms by which selective tmTNF overexpression leads to SpA-like pathology, *tmTNF tg* mice were crossed to either TNF-RI knockout or TNF-RII knockout mice, monitored for disease symptoms, and analyzed histologically at the age of 100–120 d. Peripheral synovitis, osteitis, and enthesitis were observed in 100% of the *tmTNF^+/WT^* (20/20) as well as in 100% of the *tmTNF^+/WT^xTNF-RII^−/−^* mice (7/7) but not in the *tmTNF^+/wt^xTNF-RI^−/−^* mice (0/9; *n* > 20; *n* = 7, and *n* = 9 respectively; Fig. 7 A), confirming the findings of Alexopoulou et al. (1997) that tmTNF-mediated synovitis induction requires the presence of the TNF-RI. Cellular infiltrates at the edge of the intervertebral unit in the connective tissue as characteristic for spondylitis were also only observed in all *tmTNF^+/WT^ tg* mice as well as all *tmTNF^+/WT^xTNF-RII^−/−^* mice, and not in the *tmTNF^+/wt^xTNF-RI^−/−^* mice (Fig. 7 B). Similarly, hunchback formation and crinkled tails were observed in the *tmTNF^+/WT^* and the *tmTNF^+/WT^xTNF-RII^−/−^* mice but not in the *tmTNF^+/WT^xTNF-RI^−/−^* mice (Fig. S3 D). Surprisingly, new bone formation was not observed during histological analysis of the joints of *tmTNF^+/WT^xTNF-RII^−/−^* mice, suggesting that TNF-RII may play a specific role in the pathological osteoproliferation (Fig. 7 C). To confirm these data, we conducted a second independent experiment with detailed staining and semi-quantitative scoring of axial and peripheral joints (Fig. 7 D). Although low scores of osteoproliferation were observed in 5% and 27% of peripheral joints and spines, respectively, of the *tmTNF^+/WT^xTNF-RII^−/−^* animals, this was lower than in the *tmTNF tg* animals (osteoproliferation in 25% and 75% of peripheral joints and spines, respectively). Collectively these data indicate that, whereas TNF-RI is essential for inflammation, TNF-RII signaling contributes to pathological new bone formation triggered by tmTNF overexpression.

### Expression of tmTNF by stromal cells is required and sufficient for the induction of SpA-like disease

**Figure 8.**
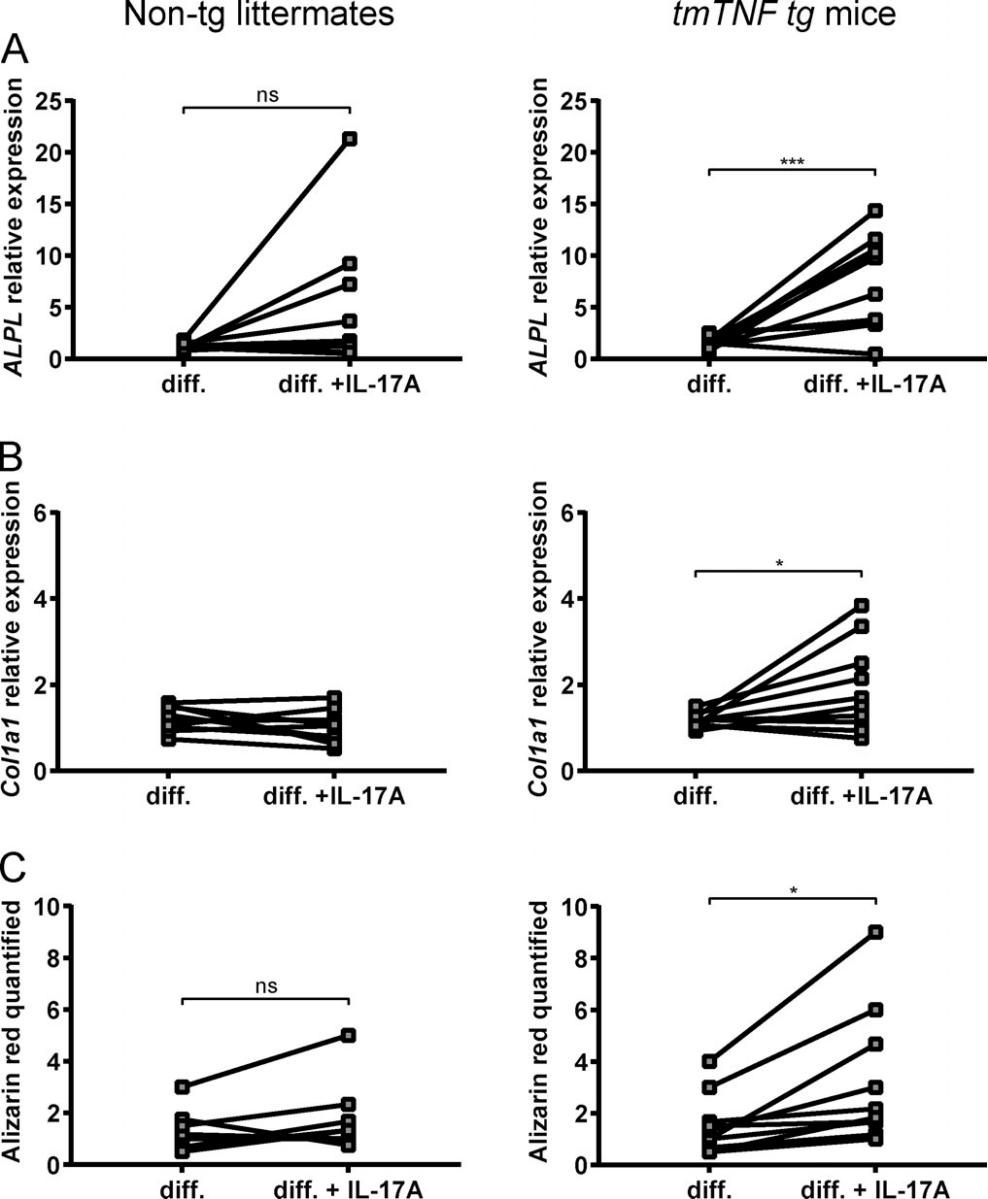
**tmTNF overexpression on calvarial fibroblasts enhances osteoblast differentiation in an inflammatory environment.** Calvarial fibroblasts from *tmTNF tg* mice and nontg littermates were differentiated (diff.) toward osteoblasts in osteogenic differentiation medium with or without rIL-17A. **(A)** Relative alkaline phosphatase mRNA expression, measured by quantitative real-time PCR at day 7 after differentiation in respectively nontg littermates and *tmTNF tg* mice. **(B)** Relative *Col1a1* mRNA expression, measured by quantitative real-time PCR at day 7 after differentiation in, respectively, nontg littermates and *tmTNF tg* mice. **(C)** Quantification of Alizarin red staining for mineralization at day 28 after differentiation in, respectively, nontg littermates and *tmTNF tg* mice. The P value was determined by an unpaired *t* test (A, B, and C) or a Mann–Whitney test (A and C). Values depicted are means ± SEM; *n* = 9 or 10 mice per group, representative of two independent experiments; *, P < 0.05; ***, P < 0.001; ns, not significant.

Based on the identification of a specific stromal signature in SpA synovitis (Yeremenko et al., 2013), we have recently postulated that stromal cells may play a crucial role in SpA immunopathology. To explore this hypothesis in the context of tmTNF overexpression, we investigated first whether tmTNF overexpression on calvarial fibroblasts leads to enhanced differentiation toward osteoblasts. Fibroblasts derived from skulls from either *tmTNF tg* mice or nontg littermates were cultured and differentiated with osteogenic medium with or without IL-17A, as an additional pro-inflammatory cytokine, for a maximum of 27 d. 7 d after differentiation, mRNA levels were measured for genes regulating osteogenesis. Alkaline phosphatase expression was significantly increased in the *tmTNF tg* mice in osteogenic medium with additional IL-17A, which was not the case in the nontg littermates (Fig. 8 A). Collagen type I revealed a similar increased expression in the *tmTNF tg* cells in osteogenic medium with additional IL-17A, whereas there was no increase observed in collagen type I expression in nontg cells (Fig. 8 B). Mineralization was also analyzed at day 21 (data not shown) and day 28 (Fig. 8 C) with an Alizarin red staining. Significantly more mineralization was measured with additional IL-17A in tmTNF-overexpressing cells compared with nontg cells.

**Figure 9.**
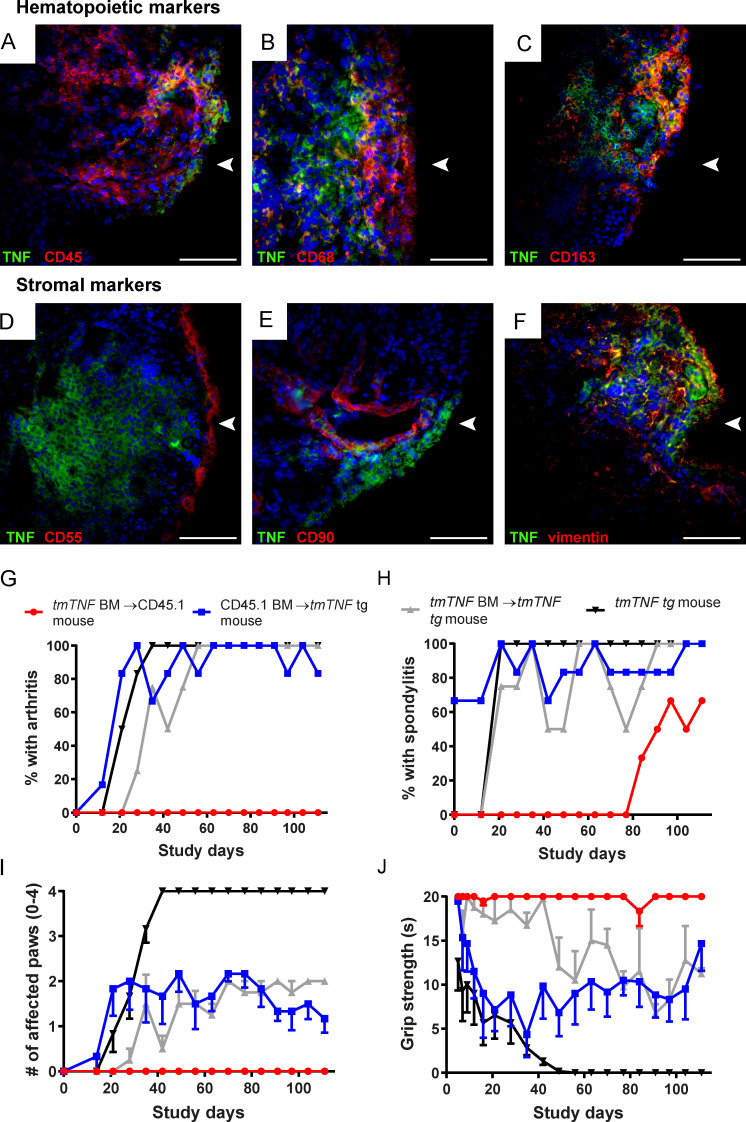
**Stromal overexpression of tmTNF is sufficient for both arthritis and spondylitis induction. (A–F)** Representative photographs of double immunofluorescence staining of TNF (green) and, respectively, CD45, CD68, CD163, CD55, CD90, and vimentin in red. Nucleus staining was performed with DAPI (blue). *n* = 5 SpA patients analyzed. Arrowhead indicates lining layer. Scale bars, 100 µm. **(G–J)** Clinical scores of BMT experiment in *tmTNF tg* and CD45.1 congenic WT mice. **(G)** Percentage with arthritis in the four groups over time. P < 0.05 for hematopoietic tmTNF expression group compared with all other groups for every time point after 60 study days. **(H)** Percentage of spondylitis in the four groups over time. P = 0.033 for hematopoietic tmTNF expression group compared with all other groups at 60 d. **(I)** Number of affected paws per animal per group. P < 0.01 for hematopoietic tmTNF expression group (red circles) compared with all other groups. **(J)** Grip strength in seconds over time is depicted. P < 0.05 for hematopoietic tmTNF expression group (red circles) compared with all other groups. The P value was determined by a Fisher’s exact test for each time point (G and H). Kruskal–Wallis test followed by Dunn’s multiple comparison (I and J). Values are mean ± SEM; *n* = 6 per group; P values < 0.05 are considered statistically significant. BM, bone marrow.

To explore whether stromal tmTNF expression may also be relevant to SpA pathobiology in vivo, we first performed double immunofluorescence stainings on SpA synovial tissue sections to assess whether TNF was expressed on the cell membrane of not only synovial macrophages but also FLSs. tmTNF expression was indeed observed on CD45^+^ hematopoietic cells, CD68^+^ and CD163^+^ macrophages (Fig. 9, A–C) but also on vimentin-positive stromal cells in the synovial lining layer (Fig. 9 F). tmTNF did not colocalize with either CD55 or CD90 stromal markers (Fig. 9, D and E). In contrast to the tmTNF/sTNF balance, which is specifically altered in SpA versus RA (Fig. 1), the expression of tmTNF by both hematopoietic and stromal cells as such is not specific for SpA, as similar double stainings were obtained with RA synovium (data not shown). To formally address the potential role of the stromal tmTNF overexpression, we made bone marrow chimeric mice overexpressing tmTNF either only on hematopoietic cells or only on radio-resistant stromal cells. Non-treated *tmTNF tg* mice and lethally irradiated *tmTNF tg* mice reconstituted with their own *tmTNF tg* bone marrow were used as controls. Bone marrow reconstitution was between 70–90% and 90–95%, respectively, 4 and 8 wk after bone marrow transplantation (BMT; data not shown). In line with our previous experiments, all nontreated *tmTNF tg* animals developed arthritis as well as spondylitis at the mean age of 9 and 6 wk, respectively (Fig. 9, G and H). *tmTNF tg* mice that were irradiated and rescued with *tmTNF tg* BMT (*tmTNF tg* BM→*tmTNF tg* mouse, “treated control” group) also developed arthritis and spondylitis with similar time of onset and incidence (Fig. 9, G and H), albeit the severity of the peripheral arthritis as assessed by the number of affected paws (Fig. 9 I) and grip strength (Fig. 9 J) was decreased compared with the untreated control group (*tmTNF tg* mouse). Interestingly, animals that only overexpress tmTNF on stromal cells, thus irradiated *tmTNF tg* mice rescued with WT BMT (CD45.1 BM→*tmTNF tg* mouse), developed arthritis as well as spondylitis with the same disease onset rate as both control groups (Fig. 9, G and H) and similar peripheral disease severity as the treated control group. In sharp contrast, mice with selective overexpression of tmTNF on hematopoietic cells (*tmTNF tg* BM→CD45.1 mouse) were completely protected from peripheral joint disease (Fig. 9, G, I, and J; P < 0.05 in Fig. 9, I and J) and displayed a lower incidence (66.7% versus 100%) and delayed onset (87.5 d) of spondylitis in comparison with the treated control group (12 d; P = 0.033 at 60 d; Fig. 9 H). Collectively, these data indicate that tmTNF overexpression on stromal cells is required and sufficient for the full articular SpA phenotype in this model, whereas hematopoietic tmTNF expression can induce some spondylitis but no peripheral arthritis within the 16 wk the animals were followed for disease development.

## Discussion

TNF is one of the best studied and most important pro-inflammatory cytokines. It plays a major role in many chronic inflammatory diseases, including RA and inflammatory bowel disease, as evidenced by experimental overexpression of TNF in different rodent models as well as by targeted TNF blockade in patients (McInnes and Schett, 2011). The cellular and molecular mechanisms contributing to TNF-driven chronic tissue inflammation have been well delineated (Apostolaki et al., 2010; Kollias, 2005), as well as focal tissue destruction. In chronic arthritis, TNF directly contributes to focal cartilage and bone damage by stimulating the production of destructive enzymes, by triggering the activation of osteoclasts, and at the same time inhibiting bone and cartilage repair pathways (Blüml et al., 2014; Diarra et al., 2007; Ritchlin et al., 2003).

What is not yet fully understood, however, is by which mechanisms the same cytokine could contribute to inflammatory disorders with completely different phenotypes, pathologies, and structural damage. A prototypical example is SpA, a highly TNF blockade–responsive form of chronic inflammatory arthritis that displays a set of unique characteristics such as spinal involvement, tendinitis, osteitis, and extensive new bone formation that are not recapitulated in the commonly used TNF overexpression models (Dougados and Baeten, 2011; Vieira-Sousa et al., 2015). Whereas this paradox may be partially explained by the interaction of TNF with other pro-inflammatory cytokines in each specific disease setting, such as IL-6 in RA versus IL-17A in SpA (Schett et al., 2013; Yeremenko et al., 2014), the present study proposes a new concept that the exact expression form of a single cytokine, in this case soluble versus tmTNF, may also contribute to determine the phenotype and pathology of a disease.

Two previous studies reported mild peripheral arthritis and spinal deformities in the *tmTNF tg* mice, indicating that tmTNF is sufficient to drive chronic arthritis and spondylitis (Alexopoulou et al., 1997; Edwards et al., 2006). However, these studies neither pursued the hypothesis that tmTNF may be responsible for osteoproliferation, the hallmark of SpA, nor mechanistically linked the phenotypic observation in the *tmTNF tg* mice to the pathobiology of human SpA. In sharp contrast with the destructive polysynovitis observed in the TNF overexpression models with high sTNF production (Keffer et al., 1991; Kontoyiannis et al., 1999), we show here that selective overexpression of tmTNF induces a distinct phenotype with a 100% incidence of spinal as well as peripheral joint inflammation in the absence of signs of severe systemic inflammation. Detailed histopathology confirmed the previously reported synovial inflammation in these mice (Alexopoulou et al., 1997), but additionally revealed marked enthesitis and osteitis, which are both key pathological features of human SpA. Whereas synovial inflammation in proximity of the enthesis has been reported in the *TNF^ΔARE^* mice (Jacques et al., 2014; Kontoyiannis et al., 1999), the *tmTNF tg* mice showed marked infiltration of the enthesis itself with leukocytes. In addition to the peripheral pathology, histological analysis confirmed spinal enthesitis and osteitis, again features that are reminiscent of human SpA and have not been observed in the sTNF-overexpressing models. Most strikingly, however, selective tmTNF overexpression mimicked not only the pathology of SpA (synovitis as well as peripheral and axial enthesitis and osteitis) but also the structural phenotype of this disease. Both peripheral joints and spine showed focal cartilage and bone destruction, which appears relatively mild in comparison with sTNF overexpression (Keffer et al., 1991). More notable is the marked endochondral new bone formation ultimately leading to complete joint ankylosis over time. In line with previous observations, the pathological new bone formation was not merely a repair mechanism occurring after resolution of inflammation, as we observed foci of hypertrophic chondrocytes only in close proximity of articular tissue inflammation (Rudwaleit et al., 2009; Turina et al., 2014; van Duivenvoorde et al., 2012). Collectively, the clinical, pathological, and radiological assessment of the model indicates that selective overexpression of tmTNF recapitulates the key articular features of human SpA. Interestingly, *tmTNF tg* mice, which overexpress tmTNF systemically in all compartments, did not spontaneously develop clinical or subclinical extra-articular disease of gut, eyes, or skin. This observation suggests that, albeit we do not have information on TACE activity and tmTNF/sTNF expression in these organs in human SpA, a potential disbalance toward tmTNF expression is unlikely to be sufficient to drive these disease manifestations. One potential explanation is that tmTNF overexpression may confer increased susceptibility to extra-articular inflammation, but additional triggers are needed to induce full-blown colitis, uveitis, and/or psoriasis. Supporting a role for tmTNF in extra-articular manifestations, recent studies in human Crohn’s disease indicated that tmTNF expression in the gut is a good predictor of response to TNF inhibition (Atreya et al., 2014) and that binding of anti-TNF antibodies to tmTNF activates a macrophage-mediated immunoregulatory response that is crucial for the therapeutic effects in inflammatory bowel disease (Levin et al., 2016a; Levin et al., 2016b). Alternatively, however, either sTNF, as suggested by the gut inflammation in the *TNF^ΔARE^* mice (Armaka et al., 2008; Jacques et al., 2014; Kontoyiannis et al., 1999), or other cytokines may be more important than tmTNF for these extra-articular manifestations of SpA.

The striking difference in pheno- and pathotype between the tmTNF tg mice and other TNF overexpression models with high sTNF levels raises the question how these two forms of TNF can induce distinct biological responses. Previous in vitro and in vivo work convincingly demonstrated that the functions of sTNF and tmTNF are not completely overlapping. Among other examples, sTNF but not tmTNF strongly promotes osteoclastogenesis in vitro (Abu-Amer et al., 2000), whereas tmTNF but not sTNF actively suppresses autoimmune inflammation in experimental autoimmune encephalomyelitis (Alexopoulou et al., 2006). One potential explanation for these differential effects is the difference in relative affinity of sTNF versus tmTNF for the TNF receptors: whereas both sTNF and tmTNF trigger TNF-RI, tmTNF was reported to have a higher affinity for TNF-RII (Campbell et al., 2003; Grell et al., 1995). As TNF-RI has been shown to be crucial for RA-like polysynovitis in other TNF overexpression models (Diarra et al., 2007; Kontoyiannis et al., 1999), we tested whether TNF-RII may be more important than TNF-RI in the SpA-like phenotype of *tmTNF tg* mice. In line with a previous study showing that tmTNF-mediated peripheral synovitis requires the presence of TNF-RI (Alexopoulou et al., 1997), we did not observe any arthritis or spondylitis in *tmTNF^+/WT^xTNF-RI^−/−^* mice. In contrast, *tmTNF^+/WT^xTNF-RII^−/−^* mice developed clinical spondylitis as marked by hunchback formation and a crinkled tail and subclinical arthritis with synovitis, enthesitis, and osteitis in the ankle joints. Interestingly, the *tmTNF^+/WT^xTNF-RII^−/−^* mice examined in these experiments displayed less endochondral new bone formation than *tmTNF^+/WT^* mice at the age of 100–120 d. In agreement with the fact that peripheral arthritis is delayed in the absence of the TNF-RII (Alexopoulou et al., 1997), these data suggest a complex interplay between TNF-RI and TNF-RII in the pathology of *tmTNF tg* mice. Finally, it needs to be noted that this study focused on focal erosions and new bone formation in axial and peripheral joints and was not designed to assess systemic bone loss. More detailed imaging and biomarker studies at different time points remain warranted to fully assess the systemic bone phenotype of *tmTNF tg* mice.

Besides differential activation of TNF-RI versus TNF-RII, a second possible explanation for the distinct phenotype of *tmTNF tg* mice is the expression by specific cell types, especially since tmTNF has also been suggested to be involved in outside-in signaling (Eissner et al., 2000, 2004). In central nervous system inflammation, for example, pathology was shown to be mediated by astrocyte-specific, but not neuron-specific, tmTNF expression (Akassoglou et al., 1997). As stromal cells have been recently proposed to play a major role in human and experimental SpA (Armaka et al., 2008; Yeremenko et al., 2013), we aimed to investigate the effect of tmTNF overexpression on stromal cells. In the current study, we show that tmTNF overexpression on mouse calvarial cells with additional IL-17A leads to enhanced alkaline phosphatase and collagen type I expression and an increased mineralization compared with nontg calvarial cells, suggesting a direct effect of tmTNF on new bone formation. Other factors could contribute as well, such as a synergistic effect of TNF and IL-17A (Osta et al., 2014), which is also present in the in vivo model. The potential relevance of these in vitro findings was highlighted by the fact that not only synovial macrophages but also vimentin-positive stromal cells of the inflamed human SpA synovium express tmTNF, indicating that both cell types can contribute to the pathobiology of SpA. Importantly, this is not unique to SpA as both cell types also express tmTNF, albeit at a much lower level, in RA synovitis. The fact that tmTNF expression is higher in SpA on both hematopoietic and stromal cells is consistent with the observation of impaired TACE activity in both synovial fibroblasts and hematopoietic cells of SF and peripheral blood, but raises at the same time the question of the relevance of both cell types in the pathobiology of SpA. To study the relative contribution of stromal versus hematopoietic tmTNF overexpression in vivo, we made bone marrow chimeric mice overexpressing tmTNF either on hematopoietic cells or on radio-resistant stromal cells. Mice overexpressing tmTNF on hematopoietic cells were completely protected from peripheral joint disease and displayed a lower incidence and delayed onset of spondylitis, whereas mice overexpressing tmTNF on stromal cells developed both spondylitis and arthritis with the same onset and severity as the control-treated group. Radioresistant anti-inflammatory macrophages have been described in the skin (Barreiro et al., 2016) and show similarity to self-renewing synovial lining layer macrophages (Culemann et al., 2019). Whereas it cannot be formally excluded that sub-lethal irradiation spares not only stromal cells but also radioresistant myeloid cells, the disease suppression in animals expressing tmTNF on hematopoietic cells clearly indicates the specific role of stromal tmTNF expression. Interestingly, mice overexpressing tmTNF on hematopoietic cells and developing delayed spondylitis showed a destructive rather than remodeling phenotype on radiographical examination and histology (data not shown). This observation is in line with a previously described SpA-like animal model based on IL-17A, which proposes a role for macrophages in bone destruction (Adamopoulos et al., 2015). These data indicate that tmTNF overexpression on stromal cells plays an essential role in this model in general and in the new bone formation in particular.

A third key question raised by the observations in the *tmTNF tg* mice is to what extent stromal tmTNF expression is also relevant to human SpA. Making use of synovial tissue biopsies, which is the only reasonably accessible target tissue in human SpA, we demonstrated a relative overexpression of tmTNF over sTNF in active SpA versus active RA as control at similar TNF mRNA expression levels and RNA stability (data not shown). The fact that not only sTNF but also sTNF-RI, sTNF-RII, and sCD163 were decreased in SpA versus RA SF indicates that impaired ADAM17 (or TACE) expression and/or activity might be implicated in the relative overexpression of tmTNF over sTNF in the SpA synovial lining layer. Although we did not observe differences in mRNA and protein levels of ADAM17 in SpA versus RA synovitis, we were able to show a clear decrease in enzymatic activity of ADAM17 in SpA FLS compared with RA FLS, indeed suggesting impaired ADAM17 activity. Additional analysis of PBMCs confirmed that ADAM17 activity was reduced in SpA rather than increased in RA, and indicated that this is a systematic feature of SpA rather than a local epiphenomenon in the inflamed joint. At this point, the reason for decreased ADAM17 activity in SpA synovitis remains elusive. Protein expression and function of ADAM17 are complex (Zunke and Rose-John, 2017) and regulated at several steps (Schlöndorff et al., 2000; Willems et al., 2010; Adrain et al., 2012; McIlwain et al., 2012). Dysfunctional ADAM17 needs to be studied in the context of tissue inflammation and new bone formation in SpA.

Collectively, the human and animal data of the present study concord to indicate that TNF can drive strictly distinct inflammatory pathologies depending on its specific expression form. We propose that the tmTNF rather than sTNF contributes to key pathological features of SpA, including new bone formation. Further elucidation of the mechanisms underlying the increased tmTNF expression as well as the tmTNF-induced pathology not only will help us to understand how a single cytokine can drive distinct pathologies but also may have direct therapeutic implications for the treatment of SpA. Despite the success of TNF inhibition in this disease, this therapeutic approach still fails to induce full inflammatory remission in a majority of patients, and even in those with inflammatory remission, the progression of structural damage in form of new bone formation is not halted (van der Heijde et al., 2008a, 2008b; Baraliakos et al., 2014). The current findings raise the hypothesis that current anti-TNF drugs may efficiently neutralize sTNF without sufficiently blocking tmTNF, especially in poorly vascularized stromal compartments such as the enthesis. If this hypothesis is correct, alternative strategies to target tmTNF expression, TNF-R signaling, and/or downstream effector mechanisms may open new avenues for effective treatment of inflammation and structural progression in SpA.

## Materials and methods

### Patients

This study included 64 patients who fulfilled the European Spondylarthropathy Study Group criteria for SpA (Dougados et al., 1991) and 60 patients who fulfilled the American College of Rheumatology/European League Against Rheumatism 2010 criteria for RA (Aletaha et al., 2010). All patients had active disease with effusion of at least one knee joint, and none of the patients was treated with a biological agent. Synovial biopsy samples were obtained by needle arthroscopy from actively inflamed joints (Baeten et al., 1999) of 20 SpA patients (32% female patients, mean age 40 yr, mean C-reactive protein [CRP] level 32 mg/liter, and mean swollen joint count 2.5) and 18 RA patients (58% female patients, mean age 56 yr, mean CRP level 39.5 mg/liter, and mean swollen joint count 11) for qPCR analyses, and in 20 SpA patients (37% female patients, mean age 43 yr, mean CRP level 26 mg/liter, and mean swollen joint count 2.5) and 20 RA patients (75% female patients, mean age 52 yr, mean CRP level 36.5 mg/liter, and mean swollen joint count 7) for immunohistochemistry. SF was obtained by joint puncture in 25 patients with SpA (39% female patients, mean age 40 yr, mean CRP level 22.1 mg/liter, and mean swollen joint count 2.8) and 22 patients with RA (71% female patients, mean age 58 yr, mean CRP level 24.6 mg/liter, and mean swollen joint count 8.1). SFMCs and PBMCs were obtained by Ficoll–Hypaque density gradient centrifugation. The amount of samples used per experiment was based on availability. Detailed patient characteristics are available upon request from the corresponding author. Written informed consent was obtained from all patients before inclusion in the study, which was approved by the Local Ethics Committee of the Academic Medical Center at the University of Amsterdam. No samples were excluded from analyses.

### ELISA

Levels of sTNF (Pelipair M9323; Sanquin), soluble TNF-RI (BMS203CE; eBioscience), soluble TNF-RII (BMS211CE; eBioscience), and soluble CD163 (DY1607; R&D Systems) were measured by ELISA in SF according to the instructions of the manufacturers.

### qPCR

Total RNA was extracted from synovial tissue samples according to the protocol of the RNeasy FFPE Kit (Qiagen). The quantity of the RNA was assessed by nanodrop (NanoVue Plus, General Electric), and 1–2 µg of RNA was reverse-transcribed into cDNA using a high-capacity cDNA Archive Kit (Applied Biosystems). TaqMan gene expression assays for human *GAPDH* (4310884E), *TNF* (Hs00174128_m1), *TNF-RI* (Hs01042313_m1), *TNF-RII* (Hs00961749_m1), and *ADAM17* (Hs01041915_m1) were purchased from Applied Biosystems, and gene expression was measured in duplex reactions. Gene expression in mouse samples (*GAPDH*, *ALPL*, and *Col1a1*) was measured in duplex reactions using SYBR green primers (sequences are available upon request). The relative expression (represented in arbitrary units) was calculated with the “2^(−ddCt) method,” in which dCt = Ct gene − Ct housekeeping gene and ddCt = dCt sample − dCt calibrator. *GAPDH* was used as a housekeeping gene and one of the samples as an internal calibrator (Livak and Schmittgen, 2001). The results were calculated using the StepOne Software v2.1 (Applied Biosystems).

### Immunohistochemistry

Synovial biopsy samples were snap-frozen in Tissue-Tek optimal cutting temperature compound (Miles) immediately after collection. Cryostat sections (5 µm) were cut and mounted on Starfrost adhesive glass slides (Waldemar Knittel Glasbearbeitungs). Frozen sections were acetone-fixed (10 min) and stained with 1 μg/ml mAb directed against TNF (clone 52B83; Hycult Biotech) or 5 μg/ml mAb directed against ADAM17 (clone ab57484; Abcam) overnight at 4°C. The anti-TNF antibody clone 52B83 does not bind TNF when it is captured by one of the TNF-Rs (Gerspach et al., 2000); accordingly, cellular staining reflects tmTNF expression, whereas noncellular staining indicates sTNF that is not bound to its receptors. After rinsing, sections were sequentially incubated with a biotinylated secondary antibody, a streptavidin–horseradish peroxidase link, aminoethylcarbazole substrate as chromogen (LSABII kit, Dako), and Gill’s hematoxylin as counterstain. As negative control, parallel sections were incubated with a concentration-matched mouse IgG1 isotype antibody. All samples were stained in a single run to minimize technical biases, and subsequently scored semi-quantitatively for cellular infiltration by three independent observers (L.M. van Duivenvoorde, M.N. van Tok, and D.L.P. Baeten), who were blinded to the patients’ diagnoses (Baeten et al., 2004b).

### Immunofluorescence

Frozen synovial tissue sections were fixed in acetone and blocked with 10% goat serum (Dako), followed by incubation with the Biotin blocking system (Dako). Stainings with mAb directed against TNF (clone 52B83; Hycult Biotech), CD45 (clone HI30; BioLegend), CD55 (clone JS11; BioLegend), CD68 (clone Y1/82A; BioLegend), CD90 (clone 5E10; BioLegend), CD163 (clone GHI/61; BioLegend), and vimentin (clone D21H3; Cell Signaling Technology) were performed overnight at 4°C, followed by incubation with Alexa Fluor 488/Alexa Fluor 555–conjugated goat anti-mouse and goat anti-rabbit secondary antibodies. Slides were mounted with Vectashield containing DAPI (Vector Laboratories) and analyzed on a fluorescence imaging microscope (Leica DMRA) coupled to a charge-coupled device camera, with results analyzed using Image-Pro Plus software (Media Cybernetics, Dutch Vision Components).

### ADAM17 activity assay

Activity of TACE/ADAM17 in vivo was determined in cell lysates from FLS cultures (SpA cell lines: *n* = 7 and RA cell lines: *n* = 6) using the AnaSpec SensoLyte 520 TACE Activity Assay, following the manufacturer’s instructions (AnaSpec).

### Mice

*tmTNF* transgenic mice (*TgA86*; Alexopoulou et al., 1997) and *TNF^ΔARE^* mice were kindly provided by G. Kollias, and the breeding was maintained in the Academic Medical Center animal facility. *tmTNF tg* mice were crossed back on a full C57Bl/6 background (C57Bl/6JOlaHsD). B6.SJL-Ptprc^a^Pep3^b^/BoyCrl (CD45.1) congenic mice were obtained from Charles River Laboratories. *TNF-RI^−/−^* (*Tnfrsf1a^tm1Imx^*) and *TNF-RII^−/−^* (*Tnfrsf1b^tm1Mwm^*) mice were obtained from The Jackson Laboratory. Experiments were performed in accordance with national legislation and under supervision of the Animal Experimental Committee of the Academic Medical Center and the University of Amsterdam. For our experiments, we did not use any type of randomization as the genotypes of the experimental groups differ.

### Clinical scoring

Mice were scored at least weekly for weight loss, arthritis (swelling and deformation of hind limbs and front paws), and spondylitis (hunchback formation and swelling or crinkling of the tail; van Duivenvoorde et al., 2012; van Tok et al., 2017) until 100 d of age. To measure grip strength, mice were placed on top of the lid of the cage, after which the lid was carefully turned upside down, and the time was measured that the mice held the grid (for a maximum of 20 s). No animals were excluded from analyses. Due to the clear phenotype of the *tmTNF tg* mice, it was not possible to blind the observer during scoring and evaluation of the in vivo experiments.

### Sample processing and histological staining

Paws, tail, spine, sacroiliac joints, skin, eyes, ileum, and ascending colon were harvested and fixed overnight in 4% formalin and sectioned. Joints were decalcified for at least 4 wk in osteosoft (Merck). Samples were embedded in paraffin and cut into 5-µm sections. Sections were deparaffinized and stained for 10 min with Mayer’s hematoxylin solution (Sigma-Aldrich). After rinsing with tap water and 96% ethanol, the sections were stained for 2 min with Accustain eosin solution (Sigma-Aldrich), dehydrated, and mounted in entellan (Merck). Alternatively, sections were deparaffinized and stained for 10 min with Weigert’s iron hematoxylin solution (Sigma-Aldrich). After rinsing for 10 min with tap water, the sections were stained for 5 min with Fast Green solution (Sigma-Aldrich), quickly rinsed in 1% acetic acid, and stained with 0.1% Safranin-O for 5 min, dehydrated, and mounted in entellan (Merck). Stained sections were scored by two independent observers (L.M. van Duivenvoorde and M.N. van Tok). Based on similar histological studies of human synovial tissue (Baeten et al., 2000, 2004a), sections were semi-quantitatively graded, according to the degree of lymphocyte infiltration, as normal, mild, moderate, or severe; the same was done for bone and cartilage destruction and proteoglycan staining. This quantitative analysis was performed on both hind paws and spine and tail sections. The scores were concordant in 84% of the cases. In 16% of the cases, the scores were discordant, and the average of the two scores was used. No samples were excluded from analyses.

### Histomorphometric analysis

Bone histomorphometry was performed using a microscope (Nikon) equipped with a digital camera and an image analysis system (OsteoMeasure; OsteoMetrics). The following parameters were measured: total size of the proliferation and specific of the hypertrophic chondrocyte part. Sections were scored by one independent observer (G. Schett) blinded for the specific mouse strain. No samples were excluded from analyses.

### Radiographical images

From three 8-mo-old *tmTNF tg* male mice and three nontg littermates (same age and gender), radiographical images were obtained from hind paws as well as spine and tails. Images were shot with the Senograph Essential (General Electric) at 28 kV and 20 mA for 10 s. No animals were excluded from analyses.

### Calvarial fibroblast cultures

Primary osteoblasts were isolated from calvariae from *tmTNF tg* mice or nontg littermates (*n* = 9 or 10 per group) after aseptic dissection and treatment with Collagenase II (100 U/ml, LS004174; Worthington). Cells were digested for 4 h at 37°C and afterwards cultured in DMEM supplemented with 10% FCS (Biowest), 2 mM L-glutamine (Life Technologies), 0.5 mg/ml penicillin–streptomycin (Life Technologies), 50 μg/ml gentamycin (Life Technologies), and 20 uM β-mercaptoethanol (Sigma-Aldrich). After expansion of the cells, cells were seeded 30,000 cells/well in a 24-well plate for Alizarin red staining or 150,000 cells/well in a 6-well plate for mRNA analyses. To induce differentiation, cells were cultured in StemXvivo osteogenic/adipogenic base medium (R&D Systems) supplemented with 2 mM L-glutamine, 0.5 mg/ml penicillin–streptomycin, 20 μM β-mercaptoehtanol, 50 μM ascorbic acid (Sigma-Aldrich), 10 mM β-glycerophosphatase (Sigma-Aldrich), and 50 ng/µl IL-17A (R&D Systems). No samples were excluded from analyses.

### Alizarin red staining

Differentiated cells in plates were washed with PBS, fixed with 4% formaldehyde (VWR) for 5 min, washed again with PBS (three times), stained with 3 mg/ml Alizarin red (Sigma-Aldrich) for 1 min, washed several times with tap water, and dried by air.

### Bone marrow chimera experiment

4-wk-old male *tmTNF tg* mice and 6-wk-old male CD45.1 congenic WT mice received twice a total body irradiation with 6 Gy in a 24-h interval. 1 d after the last irradiation, mice were rescued with total bone marrow cells intravenously. Total bone marrow cells were obtained from tibia and femurs of either *tmTNF tg* or CD45.1 congenic WT mice. Tibia and femurs were flushed with PBS. Cell suspensions were washed once with PBS, counted, and injected into the recipient mice (10^7^ cells/200 µl PBS containing 1% BSA). After BMT, mice were weekly scored for weight, arthritis, and spondylitis induction as described above. 4, 8, and 12 wk after BMT, blood was drawn via a small incision in the tail vein to check BMT engraftment. 16 wk after BMT, all animals were sacrificed. No animals were excluded from analyses.

### Sample size selection

For animal in vivo experiments, the program Power and Precision Pro (http://www.power-analysis.com/) and the two-sided *t* test for independent samples were used to calculate the size of the group. We planned our studies of a continuous response variable from independent control and experimental subjects with one control per experimental subject. In a previous study, the response within each subject group was normally distributed with standard deviation 5. If the true difference in the experimental and control means is 10, we will need to study five experimental subjects and five control subjects to be able to reject the null hypothesis that the population means of the experimental and control groups are equal with probability (power) 0.8. The Type I error probability associated with this test of this null hypothesis is 0.05. Also for in vitro studies, we calculated the similar sample size.

### Statistics

The D’Agostino–Pearson omnibus test was used to check data for normality. Non-parametric data of two groups were analyzed with a Mann–Whitney test. Parametric data of two groups were analyzed with an unpaired Student’s *t* test. Weight curves were analyzed with a two-way ANOVA analysis with Sidak’s multiple comparisons post-test. A log-rank (Mantel–Cox) test was performed for incidence graphs (survival) if possible. A Fisher’s exact test was performed per time point in experiments with varying incidence over time and multiple group comparisons were done with a Kruskal–Wallis test followed by Dunn’s multiple comparison. All statistical tests were two-tailed and calculated using GraphPad Prism version 8 (GraphPad Software). Values of P < 0.05 with a 95% confidence interval were considered significant.

### Data availability

All relevant data are available from the authors upon reasonable request.

### Online supplemental material

Fig. S1 shows synovial TNF expression in SpA and seronegative and seropositive RA patients. Fig. S2 shows decreased ADAM17 activity in SpA SFMCs and PBMCs compared with RA SFMCs and PBMCs. Fig. S3 shows that both *tmTNF tg* and *tmTNF^+/WT^xTNF-RII^−/−^* mice have a hunchback and crinkled tail. Fig. S4 shows a comparison of inflammation in *tmTNF tg* and *TNF^ΔARE^* mice axial and peripheral joints. Fig. S5 shows that endochondral new bone formation is only present in *tmTNF tg* mice, not in *TNF^ΔARE^* mice.
